# Ethnoracial disparities in breast cancer treatment time and survival: a systematic review with a DAG–based causal model

**DOI:** 10.1093/epirev/mxaf009

**Published:** 2025-06-06

**Authors:** Parisa M Hesari, Drexler James, Daniel J Lizotte, Greta R Bauer

**Affiliations:** Department of Epidemiology and Biostatistics, Schulich School of Medicine & Dentistry, Western University, London, ON, Canada; Department of Psychology, University of Minnesota, London, ON, Canada; Department of Epidemiology and Biostatistics, Schulich School of Medicine & Dentistry, Western University, London, ON, Canada; Department of Computer Science, Western University, London, ON, Canada; Department of Epidemiology and Biostatistics, Schulich School of Medicine & Dentistry, Western University, London, ON, Canada; Eli Coleman Institute for Sexual and Gender Health, University of Minnesota Medical School, United States

**Keywords:** racial disparity, time-to-treatment initiation, breast cancer, survival, directed acyclic graphs (DAGs)

## Abstract

For interventions aimed at redressing health disparities in breast cancer to be effective, a clear understanding of the nature and causes of these disparities is required. Our questions were: what is the current evidence for ethnoracial disparities in time-to-treatment initiation and survival in breast cancer, and how are the causal mechanisms of these disparities conceptualized in the literature? A comprehensive systematic search of studies on cohorts of female patients with breast cancer diagnosed with stage I-III was performed. Directed acyclic graphs were used to describe implicit causal relationships between racial/ethnic group membership and time-to-treatment initiation and survival outcomes. This review revealed strong evidence for ethnoracial disparities in both time to treatment and survival among patients with breast cancer. Unmeasured factors identified by the authors highlighted gaps in data sources and opportunities for causal reasoning. Although the existing literature describes ethnoracial disparities, there is very limited discussion of causal mechanisms and no discussion of system-level rather than individual-level effects. Addressing established ethnoracial disparities in breast cancer requires new research that explicitly considers the causal mechanisms of potential interventions, incorporating unmeasured factors contributing to these disparities.

**Trial registration:** PROSPERO identifier: CRD42023391901

## Introduction

Inequalities in access to care across racial/ethnic groups are a known driver of differential health outcomes.^1^ An increasing body of evidence demonstrates that communities of color have been disproportionately affected by systemic racism that leads to disparities in chronic disease outcomes, including for breast cancer.^2‑4^ Though breast cancer survival has increased globally due to advances in survivorship care, treatment modalities, and prevention methods,^4^^,^^5^ studies have shown less improved survival in marginalized groups, even after controlling for biological and other characteristics.^6^^,^^7^

There is increasing evidence that factors associated with delays in treatment initiation vary across racial/ethnic groups of patients with breast cancer and that this drives disparities in outcomes.^8‑10^ Studies examining the impact of therapy duration on survival across these groups have identified significant differences in time to treatment initiation and survival.^10^^,^^11^ Many factors affecting disparities in breast cancer have been identified, including social and health determinants along with individualized and tumor-related factors.^12‑15^ However, many studies have prioritized establishing association rather than examining the causal mechanisms underlying these associations,^16‑18^ and many studies rely on cancer registry data, which often do not contain the information needed to assess such mechanisms.^19^

Although information on unmeasured confounders remains limited, causal diagrams provide a powerful framework for advancing research on racial disparities in breast cancer treatment. The use of causal inference is essential for disentangling the complex social determinants driving these disparities. By breaking down race into its contextual components or incorporating broader socioenvironmental influences, these methods can inform effective interventions. Understanding the mechanisms that influence the time to treatment initiation can inform targeted interventions at both individual and systemic levels, potentially mitigating racial disparities and improving health outcomes.^20^^,^^21^

Despite this potential value, there has been, to date, no overarching synthesis of causal reasoning on ethnoracial disparities in treatment initiation in breast cancer. To achieve this, we construct and combine study-specific directed acyclic graphs (DAGs), producing a summary DAG showing the structure of causal logic used within the field to describe relationships among ethnoraciality and other factors and their relationship to time to treatment initiation (TTI) and breast cancer survival. The resulting knowledge allows us to both 1) describe key causal logic structures considered in existing literature, and 2) identify individual and structural factors that are known to affect access to care or ethnoracial disparities but have not been addressed in the context of breast cancer.

## Methods

This systematic review is registered in the PROSPERO International Prospective Register of Systematic Reviews (ID: CRD42023391901). This review shares some characteristics with a scoping review, particularly in its effort to establish frameworks and conceptual lenses. We designed this review fundamentally as a systematic review. The primary objective of this study was to conduct a rigorous and methodologically sound synthesis of the literature, consistent with the principles of a systematic review. The establishment of the scope and breadth of literature is a byproduct of this systematic approach, rather than the main purpose.

Our methodology included 2 primary activities. First, this review was conducted according to a specifically designed protocol that adheres to Cochrane methods.^22^ We systematically reviewed literature on ethnoracial disparities and time to treatment and/or survival, adhering to the Preferred Reporting Items for Systematic Review and Meta-Analysis Protocols (PRISMA-P)^23^ guidelines using the Covidence platform.^24^ Second, we constructed DAGs representing the presumed causal relationships among factors in each included study and consolidated them into a single DAG, following a modification of the evidence synthesis for constructing DAGs guideline.^25^

### Systematic literature review on ethnoracial disparities

#### Eligibility criteria

English language peer-reviewed studies published in academic journals were eligible for inclusion if they focused on patients aged 18 years or older diagnosed with stage I to III breast cancer between 1995 and 2019. Study participants must have undergone at least 1 form of systemic or local therapy.

In this review, studies that did not adequately consider race/ethnicity were excluded. Simply reporting race/ethnicity as a baseline characteristic was not sufficient for inclusion. To be considered, studies needed to either ensure that the sample was representative of the target population with respect to race/ethnicity or include race/ethnicity in the main statistical analysis. Additionally, studies that conducted subgroup analyses across different racial/ethnic groups were included.

Studies that restricted their analysis to a particular racial/ethnic group were only included if they contextualized their findings by comparing them with national data or to outcomes in other racial/ethnic groups. As part of the full-text screening, we assessed whether studies provided any cross-group comparison or broader contextual framing, such as referencing national trends or explicitly discussing implications for racial disparities. For example, a study focusing exclusively on Hispanic individuals was included if it analyzed within-group heterogeneity and compared findings with national trends across racial/ethnic groups. This approach ensured that included studies contributed to a meaningful understanding of racial/ethnic disparities in breast cancer, rather than presenting isolated within-group findings.

Studies that did not explicitly exclude patients with stage IV breast cancer were excluded, because treatment primarily involves palliative care. Studies during the COVID-19 pandemic were excluded due to pandemic-related changes in the treatment modalities and management of patients with breast cancer, influenced by both individual and health care system factors.

#### Outcomes: Time to treatment and survival measure

We considered 2 outcome measures: TTI and survival. Time to treatment initiation was defined as the time from diagnosis to initiation of local therapies (eg, surgery) and/or systemic therapies (eg, chemotherapy.) Survival time was measured as the interval between treatment initiation and events such as breast cancer–related deaths or follow-up time for survivors.

#### Search strategy

Eligible studies were identified in the PubMed, Ovid, Web of Science, and Cochrane Library databases. A title-filtered search was also conducted through Google Scholar using the primary keywords “breast cancer” and “time to treatment,” as was a manual search using reference lists of the included studies. The primary keywords were searched (Table S1). We did not require that the word “race” appear in the title or abstract, because this would exclude many relevant studies. Full details are provided in the protocol document.^22^

#### Study records

Duplicate records were removed, the remaining studies were screened based on title and abstract, and then full texts were assessed for inclusion. Data extraction was performed by 2 independent reviewers, and reviewers convened to discuss any uncertainties, including any errors in the data collection process or uncertainty regarding inclusion of a study. Studies with correction or retraction notices were removed.

#### Data extraction and risk-of-bias assessment

We extracted, using Covidence, study characteristics including main objective, study design, source of data, sample size, data collection period, ethnoracial group categories, types of treatments assessed, and lists of author-identified unmeasured factors.

In the creation of the comprehensive DAGs, relationships among measured factors were extracted and carefully evaluated based on the consistency and strength of the evidence across studies. Specifically, when multiple studies assessed the same factor but reported conflicting results (eg, 1 study found a positive association, whereas another found a negative or null association), we incorporated the relationship into the DAG based on the overall weight of evidence. This involved considering the quality of the studies, the study design, the sample size, and the robustness of the reported associations. If the evidence for a relationship was inconclusive or inconsistent, we critically assessed whether to include it in the DAG. For example, if 1 study found a strong inverse association between 1 factor and breast cancer outcomes whereas another found no association, we evaluated factors such as sample size, study design (eg, prospective cohort vs cross-sectional), and whether confounders were adequately adjusted for. If the weight of evidence and theoretical plausibility supported a relationship, it was included in the DAG. Conversely, if findings were highly inconsistent with no clear explanation, we excluded the relationship unless a strong biological or social mechanism justified its inclusion. We aimed to include only those relationships for which there was a reasonable degree of confidence, ensuring that the DAG reflects the most likely causal structures based on the available data. Additionally, given that much of the literature is not explicitly causal, decisions to include relationships in the DAG were made based on the plausibility of the causal linkages, informed by theoretical frameworks and the biological or social mechanisms suggested in the literature. Not all extracted relationships were automatically included; only those supported by sufficient evidence and considered relevant to the research questions were incorporated into the final DAG.

The tool to assess risk of bias in cohort studies by the CLARITY Group at McMaster University^26^ was used to evaluate potential biases in cohort studies across 8 domains: selection of participants, assessment of exposure, initial status of the outcome, matching and adjustment for confounders, assessment of prognostic factors, outcome assessment, follow-up of cohorts, and co-interventions. Each domain is assessed on a nuanced scale: “definitely yes” (low risk), “probably yes” (moderate risk), “probably no” (serious risk), and “definitely no” (high risk)—providing a structured method to assess and discuss the reliability and validity of study results in terms of bias. Two reviewers assessed the risk of bias using this tool to categorize each study as having low, moderate, or high risk of bias. In the event of discrepancies, a third reviewer was consulted.

Study-specific DAGs were initially created by 1 person and then compared and discussed with 2 other researchers to ensure consistency and reduce individual bias. Although DAGs can be subjective in their initial interpretation and creation, we took several steps to mitigate this subjectivity. First, the creation of each DAG was informed by a thorough review of the literature and existing theoretical frameworks. Second, the collaborative process of comparing and discussing the DAGs among multiple researchers helped identify and address any potential biases or discrepancies. The analysis and visualization of study-specific DAGs were performed using a combination of R packages, namely *ggdag*, *tidyverse*, *dagitty*, and *ggplot2*.

### Construction of DAGs

To synthesize causal relationships among identified factors within the included studies, we constructed: 1) a study-specific DAG for each study individually (see Figures S1-S40), and 2) a single comprehensive DAG that combined factors across all studies.

#### Study-specific DAGs

To construct study-specific DAGs, we systematically extracted factors investigated by study authors, recognizing that most of these factors were not explicitly evaluated for causal relationships. We aimed for study-specific DAGs to describe the literature (because most of that is not explicitly causal,) identify implicit causal models, and integrate causal reasoning.

#### Producing the comprehensive DAG

The comprehensive DAG was constructed based on the observed relationships between ethnoracial group membership (EGM), other variables, and the outcomes of interest: TTI and survival. The EGM variable accounts for the inclusion of diverse racial or ethnic groups. To manage the large number of other variables assessed in the literature, we grouped variables into a single node if 1) there was face validity in combining them into a broader construct, 2) if they had the same parent nodes and same child nodes, and 3) if they had similar impact and hypothesized direction along relevant causal pathways. We considered effects along 2 major causal pathways: 1) the direct causal pathway(s) from EGM to TTI and/or survival, and 2) in models that consider a mediator, the indirect causal pathway(s) related to the mediator. For instance, we would only combine income, education, and occupation into a single socioeconomic status (SES) node if their effects on relevant causal pathways align in the same direction (positive or negative) and if they had the same parents and children.

## Results

**Figure 1:**
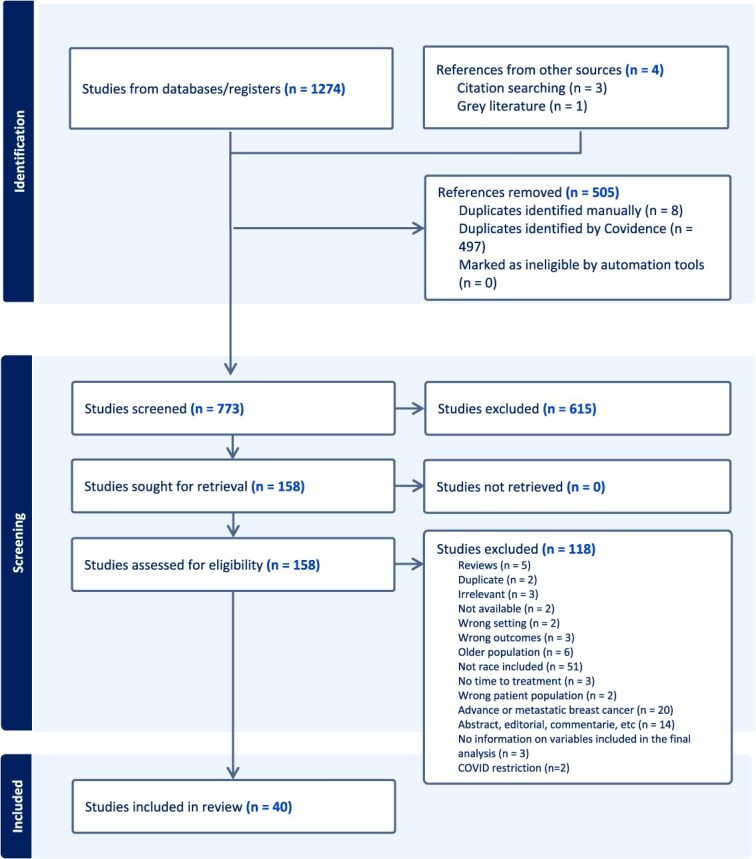
Process of identification and data extraction of studies on racial disparity and time to treatment on survival in breast cancer using the Preferred Reporting Items for Systematic Review and Meta-Analysis Protocols.

We initially identified 1278 records (Figure 1); after removing duplicates and screening, 158 studies were included for full-text review. Of these, 40 were included in the final analysis. None of the studies included in this review had correction or retraction notices. All factors assessed in the literature are summarized in Table 1, grouped according to the principles described in the ‘Producing the comprehensive DAG’ section .

**Table 1:** List of factors from included studies and detailed factors within each group.

**Factor**	**For grouped factors, individual factors studied in included articles**
Access to information	Information from family or friends, information from internet
Age	
Cancer stage	
Clinical presentation	Laterality, lymph node status (positive), lymphovascular invasion
Comorbid conditions	Comorbidity score/comorbid conditions, diabetes, heart failure, chronic pulmonary disease, cerebrovascular disease, dementia, diabetes, diabetes complications, AIDS, hemiplegia or paraplegia, liver disease, acute myocardial infarction, peptic ulcer, peripheral vascular disease, renal disease, rheumatoid disease, chronic disease, physical health care barriers
Delay in other treatments	Delay to chemotherapy
English speaking	
Facility characteristics	Academic center, hospital type, multidisciplinary care, multi-site care, facility volume, facility location, facility type, class of case (same facility for diagnosis and treatment), safety net hospital.
Financial issues	
Gender/sex	
Health risk factors	Smoking status, body mass index, family history of breast cancer
Health care discrimination	Perceived discrimination
Insurance status	Insurance, medical care source, benefit type, job loss due to cancer, transfer of care, Medicaid expansion
Marital status	
Method of diagnosis	
Physician performance	
Pretreatment care	MRI, lymph node examination, genetic testing, radiography (Initiated chemotherapy/adjuvant chemotherapy, chemotherapy with and without doxorubicin/chemotherapy type, initiated hormone therapy, endocrine therapy, type of endocrine therapy, initiated radiotherapy, contralateral prophylactic mastectomy, biologic therapy after surgery, margin status at initial surgery, preoperative MRI, additional imaging, preoperative ultrasound, biopsy, primary surgery, neoadjuvant therapy
Relapse/recurrence	Recurrence, recurrence type
Socioeconomic status	Income, education (eg, diploma), employment status, socioeconomic status
Structural barriers/access	Rural-urban, distance of health care facility, transportation, surgeon availability, (region, urban/rural, zip code, distance from medical center, metropolitan status, transportation issue, facility type (eg, surgery facility), hospital for surgery around, hospital for chemotherapy around, facility volume, access to care, available physician, military service, diagnosed cancer center, minority serving hospitals, patients treated in >1 Commission on Cancer facility, population density
Surgeon characteristics	Gender, active duty at diagnosis, performance status
Surgery as a first treatment	
Surgery type	Breast-conserving surgery, postmastectomy immediate reconstruction, postmastectomy reconstruction (time to treatment completion only), primary surgery, surgery type, postmastectomy immediate reconstruction type, reconstruction/postmastectomy immediate reconstruction, surgery decision, surveillance mammogram, in situ on index and preindex
Surgical outcomes, side effects, symptoms	Margins, pathologic complete response, wound complication
Treatment duration	Prolonged treatment duration and treatment duration
Therapy type	Chemotherapy, immunotherapy, hormone therapy, neoadjuvant therapy, radiotherapy
Trust in physician	Trust in surgeon
Tumor characteristics	Hormone receptors, ER/PR, HER2, triple negative, pathologic complete response, tumor size, tumor histology, TNM, multifocal or multicentric tumor, number of positive lymph nodes, or lymph node status/lymphovascular, lymphovascular invasion, N-stage, clinical presentation, grade, breast cancer subtype/molecular subtype
Year of diagnosis	

Abbreviations: ER, estrogen receptor; HER2, human epidermal growth factor receptor 2; MRI, magnetic resonance imaging; PR, progesterone receptor.

### Study characteristics

A list of included studies and their key characteristics is provided in Table 2. Of the 40 studies, 12 indicated that examining disparity was a primary objective. Studies categorized racial/ethnic groups in a variety of ways, mainly to support comparing Black and White populations.^21^^,^^27‑34^ Several studies also incorporated data on individuals of Hispanic and non-Hispanic ethnicity.^35^^,^^36^ Many studies examined joint racial/ethnic categories, considering combinations of racial groups with Hispanic and non-Hispanic characteristics separately, such as Black non-Hispanic, in their analysis of race/ethnicity.^36‑45^ All included studies met the criteria for low to moderate risk of bias according to the CLARITY tool.

**Table 2:** Descriptive characteristics of publications on time to treatment initiation across racial/ethnic groups.

**Reference**	**Study objective(s)**	**Study design and source of data**	**Sample size (data collection period)**	**Race categories, time to treatment(s)**	**Outcomes**	**Results or conclusion**
Hershman et al, 2005^21^	To assess racial disparities in treatment and survival	Retrospective cohort study/The Henry Ford Health System	472 (1996-2001)	Race (White, Black)/time to treatment (on time, delayed)	All-cause mortality and time to treatment completion	Black patients were more likely to delay starting chemotherapy, which was associated with poorer survival outcomes.
Alderman et al, 2010^34^	To evaluate the impact of postmastectomy breast reconstruction on the timing of chemotherapy	Retrospective cohort study/National Comprehensive Cancer Network institutions	3643 (1997-2003)	Race/ethnicity (White, Hispanic, Black, and other)/time from surgery to chemotherapy	Time from surgery to chemotherapy	Black patients had lower timing of postoperative adjuvant chemotherapy compared with White patients.
Fedewa et al, 2011^27^	To examine the relationship between race and treatment delay	Retrospective cohort/National Cancer Database (NCDB)	5 250 007 (2003-2006)	Race (White, Black, Hispanic, other)/time to treatment initiation: time to surgery	Time to treatment initiation (surgery/not surgery)	Black and Hispanic patients had higher risks of 30-, 60-, and 90-day treatment delay compared with White patients.
Bradley et al, 2012^46^	To determine whether safety net and nonsafety net hospitals influence inpatient breast cancer care	Retrospective cohort study/Virginia Cancer Registry and Virginia Health Information discharge data	3272 (1999-2005)	Race (Black and White)/time to surgery	Surgery wait time	No changes in hospital type were found across racial groups. The time between diagnosis and surgery was longer in safety net hospitals for all patients, regardless of insurance source or race.
Mosunjac et al, 2012^47^	Time to surgery in breast cancer	Retrospective chart review/a public and a private university hospital in Atlanta, GA	270 (2004-2008)	Race (Black, White, and other races)/time to breast-conserving surgery	Time to surgery	The distribution of race in public and private hospitals were statistically different.
Barry et al, 2014^48^	To analyze factors affecting timing of adjuvant chemotherapy	Matched case control/The University of Louisville School of Medicine’s James Graham Brown Cancer Center	70 (2004-2009)	Race (White, Black, and other race)/time to chemotherapy	Time to chemotherapy	No significant effect of race/ethnicity was observed.
Liederbach et al, 2015^49^	Wait times for breast surgical operations	Retrospective cohort study; the NCDB	819 175 (2003-2011)	Race (White, Black, Hispanic, Asian, and Pacific Islander)/time to surgery	Time to first surgery	Black patients’ wait time to surgery was longer than for other racial groups.
Chandwani et al, 2014^35^	To examine the role of preoperative magnetic resonance imaging on time to surgery	Retrospective cohort study/The Women’s Circle of Health Study	609 (2005-2010)	Race (White and Black (mastectomy and breast-conserving surgery)	Time to surgery	Significant differences between race and time to surgery were found. On average, Black patients received therapy later than did White patients.

**Table 2:** Continued

**Reference**	**Study objective(s)**	**Study design and source of data**	**Sample size (data collection period)**	**Race categories, time to treatment(s)**	**Outcomes**	**Results or conclusion**
Sheppard et al, 2015^36^	To investigate racial disparities in time to receiving first surgical treatment	Retrospective cohort study/hospitals in Washington, DC, and Detroit, Michigan	290 (2006-2011)	Race (White or Black)/time to surgery	Time to surgery	Prolonged delays to definitive surgery persisted among Black women. The 90-day interval has been associated with poorer outcomes.
Chavez-MacGregor et al, 2016^50^	To identify the determinants in delayed chemotherapy initiation	Retrospective cohort study/Central Cancer Registry (CCR) database	24 843 (2005-2010)	Race (NHW, NHB, Hispanic, Asian or Pacific Islander, non-Hispanic American Indian or other or unknown)/time from surgery and the first dose of chemotherapy	Overall survival, breast cancer–specific survival, and time to chemotherapy	Delays in time to chemotherapy were higher in patients of Hispanic ethnicity or NHB race compared with NHW patients.
Sanford et al, 2016^37^	To investigate the relationship between time interval from neoadjuvant chemotherapy to surgery and survival outcomes	Retrospective cohort study/The University of Texas MD Anderson Cancer Center	1101 (1995-2007)	Race: White, Black, Hispanic, other race/time from neoadjuvant chemotherapy to surgery	Overall survival, recurrence-free survival, locoregional recurrence-free survival, and time from neoadjuvant chemotherapy to surgery	Time to surgery from neoadjuvant chemotherapy, overall survival, recurrence-free survival, and locoregional recurrence-free survival were statistically different across racial/ethnic groups.
Buckley et al, 2017^38^	To examine the delayed time to surgery after mastectomy on survival in rural patients	Retrospective cohort study/NCDB	90 319 (2003-2007)	Race: NHW, NHB, Hispanic, American Indian or Alaska Native, or Asian, other or unknown race/time to surgery (mastectomy with vs without reconstruction	Overall survival and time to surgery	Race/ethnicity had no effect on overall survival or time to treatments.
Jabo et al, 2018^39^	To investigate time to treatment and survival outcomes in patients undergoing immediate breast reconstruction	Retrospective/CCR	56 782 (2004-2014)	Race/ethnicity (Asian or other, Hispanic, NHB, NHW)/time from diagnosis to surgery	Time to surgery	Significant racial disparities were observed, with NHB patients experiencing longer delays to definitive surgery compared with other racial groups, particularly among those undergoing reconstruction.

**Table 2:** Continued

**Reference**	**Study objective(s)**	**Study design and source of data**	**Sample size (data collection period)**	**Race categories, time to treatment(s)**	**Outcomes**	**Results or conclusion**
Larson et al, 2018^51^	To assess the relationship between survival, time to first treatment, and time to treatment completion in stage I-III triple-negative breast cancer	Retrospective/NCDB	17 717 (2010-2011)	White, Black, Hispanic, other/time to treatment completion (surgery, chemotherapy, and radiation)	Time to treatment completion	Time to first treatment and time to treatment completion did not affect short-term survival if time to treatment completion was shorter than 18 months. Significant differences were observed across racial/ethnic groups.
Mariella et al, 2018^40^	To identify factors associated with longer treatment initiation	Retrospective/The James Graham Brown Cancer Center in Louisville	1589 (2006-2015)	Race: White, Black, and other/time interval from breast cancer diagnosis to definitive surgery	Time to surgery	Longer time interval did not appear to significantly delay adjuvant chemotherapy or influence short-term outcomes.
Jaiswal et al, 2018^33^	To examine time to diagnosis and treatment in a safety-net hospital	Retrospective cohort/Denver Health and Hospital Authority	120 (July 1, 2010-June 30, 2012)	Race: Hispanic and non-Hispanic/time from diagnosis and presentation to first treatment	Time to first treatment	Delays in the time interval between presentation and initial treatment were significantly longer in the Hispanic group compared with the non-Hispanic group.
Eaglehouse et al, 2019^41^	To evaluate the association between time-to-surgery and overall survival	Retrospective cohort study/The Department of Defense Central Cancer Registry and the MHS Data Repository	9669 (1998-2010 and to 2015)	NHW, NHB, non-Hispanic Asian, non-Hispanic other, Hispanic/time to surgery	Overall survival and time to surgery	Longer time to surgery was associated with poorer overall survival.
Eaglehouse et al, 2019^42^	To compare time to surgery in NHB and NHW women	Retrospective cohort study/Department of Defense central cancer registry	4887 (1998-2007)	Race: NHW and NHB/time to surgery	Time to surgery	Surgical delays did not appear to explain observed racial disparities in survival.
Hoppe et al, 2019^43^	To evaluate if racial disparities persist in the treatment of patients with stage I breast cancer	Retrospective cohort study/NCDB	546 351 (2004-2014)	NHW and NHB/time to first treatment, time to surgery, chemotherapy, radiation, and endocrine therapy	Time to surgery	Black women experienced significantly longer times for time to first treatment compared with White women.
Kupstas et al, 2019^52^	To evaluate the impact of surgical treatment type on time to adjuvant chemotherapy and impact of treatment delay on survival	Retrospective cohort study/NCDB	172 043 (2010-2014)	Race: White, Black, other, unknown race; ethnicity: Spanish or Hispanic origin, no Spanish/Hispanic origin, missing/time to surgery and chemotherapy	Time to surgery and time to adjuvant chemotherapy	There was a significant association between race and time to adjuvant chemotherapy among patients who undergone surgery.

**Table 2:** Continued

**Reference**	**Study objective(s)**	**Study design and source of data**	**Sample size (data collection period)**	**Race categories, time to treatment(s)**	**Outcomes**	**Results or conclusion**
Emerson et al, 2020^28^	To evaluate association of race and age with time to treatment and treatment duration	Retrospective cohort/data from Carolina Breast Cancer Study	2841 (2008-2013)	Race (White and Black)/time to treatment initiation	Surgery, surgery + radiation, surgery+ chemotherapy, surgery+ radiation+ chemotherapy	Black women more frequently experienced delayed treatment and longer treatment duration compared with White patients. Low SES was linked to treatment delay among White women, but Black women faced high treatment delays regardless of SES.
Sutton et al, 2020^44^	To evaluate the effect of time to surgery on postoperative complications	Retrospective/the Legacy Health System Tumor Registry	463 (2011-2017)	Race (White and others)/time to surgery	Time to surgery and survival-related outcomes, which did not include race in the model	Long delays in surgery after neoadjuvant chemotherapy for breast cancer appeared to lead to worse outcomes, likely due to increased residual cancer burden over time.
Sutton et al 2020^45^	To investigate the relationship between time to surgery on residual cancer burden score and oncologic outcomes	Retrospective/the Legacy Health System Tumor Registry	392 (2011-2016)	Race (White, Asian, Hispanic, Black)/time to surgery	Time to surgery	No significant differences were found across race/ethnicity groups.
Prakash et al, 2021^53^	To determine factors associated with delays in time to surgery	Retrospective/NCDB	693 469 (2004-2014)	Race: NHW, NHB, Hispanic, other race/time to surgery	Time to surgery and time to systemic therapy	Time to surgery was influenced by the type of surgery, race/ethnicity, and insurance. Longer time to surgery was linked to poorer overall survival only for patients who had upfront surgery.
Pratt et al, 2021^54^	To examine the association between the time from diagnosis to completion of treatment modalities and survival	Retrospective/NCDB	28 284 (patients with newly diagnosed breast cancer in 2010)	Race: White, Black, Hispanic, other race or unknown/time to treatment completion: neoadjuvant chemotherapy and surgery	Time to treatment completion	Black patients experienced significantly more weeks to treatment completion and higher hazard ratios compared with other groups when measuring time to treatment completion.

**Table 2:** Continued

**Reference**	**Study objective(s)**	**Study design and source of data**	**Sample size (data collection period)**	**Race categories, time to treatment(s)**	**Outcomes**	**Results or conclusion**
Jackson et al, 2021^55^	To examine racial differences in receipt of low-value surgical care and time to surgery at high-volume hospitals	Retrospective/NCDB	378 499 (2010-2016)	Race: NHB and NHW/time to surgery was days from biopsy to surgery	Time to surgery	NHB patients treated at high-volume hospitals had higher rates of surgical delay but were less likely to undergo low-value surgical procedures compared with NHW women.
Dankwa-Mullan et al, 2021^56^	To describe clinical and nonclinical factors associated with time to surgical intervention	Retrospective study/MarketScan Commercial and Medicare Supplemental Databases	53 060 (2012-2018)	Race: White, Black, Asian, Hispanic, other race/time to surgery (breast conserving surgery vs mastectomy)	Time to mastectomy and time to breast conserving surgery	No significant differences were observed across racial/ethnic groups.
Blazek et al, 2021^57^	To examine demographics and clinical factors affecting time to treatment for patients with breast cancer who got a second opinion	Retrospective cohort/data from 8 academic, urban, and community hospitals in Columbia and Maryland	307 (2017-2019)	Race: Asian, Black, Hispanic, other race, White, unknown race/time to mastectomy from diagnosis and time to mastectomy from last chemotherapy	Time to chemotherapy and time to surgery	Low-income, Black, and Latina patients waited longer for treatment. Black patients also experienced delays between diagnosis and surgery compared with White patients.
Chagpar et al, 2022^58^	To determine factors affecting time to surgery to identify potential modifiable factors to improve timeliness of care	Retrospective using data from 2 randomized controlled trials involving 10 centers across the United States	583 (2011-2013 and 2016-2018)	Race: White, Black, Asian, others; ethnicity: Hispanic, non-Hispanic, unknown ethnicity/time to surgery (partial mastectomy): time to surgery (from core needle biopsy to definitive surgery)	Time to surgery	Patient race did not affect timeliness of care, but patients of Hispanic ethnicity were significantly less likely to have had a time to surgery <1 month.
Navarro et al, 2022^59^	To examine disparities in delays of breast cancer surgery among Asian ethnic subgroups	Retrospective/CCR	106 441 (2012-2017)	Race: Asian Indian/Pakistani, Chinese, Filipino, Hispanic, Japanese, NHB, NHW, other race, other Asian, Vietnamese/time to surgery	Time to surgery: delays of breast cancer surgery	Hispanic, Black, and some Asian ethnic groups waited longer for breast cancer surgery compared with White patients. However, Chinese patients were an exception and tended to receive surgery sooner than White patients.

**Table 2:** Continued

**Reference**	**Study objective(s)**	**Study design and source of data**	**Sample size (data collection period)**	**Race categories, time to treatment(s)**	**Outcomes**	**Results or conclusion**
Schermerhorn et al, 2022^29^	To quantify the contribution of mediators that may explain racial/ethnic disparities in breast cancer treatment delays	Retrospective/NCDB	1 349 715 (2004-2017)	Race: Black, Hispanic, White, and other non-White; time to treatments	Time to treatment	Black, Hispanic, and other non-White patients with breast cancer experienced longer treatment delays compared with White patients.
Zaveri et al, 2022^30^	To explore whether receiving care at a comprehensive breast center could mitigate disparities in time to treatment	Retrospective/National Cancer Institute-designated cancer center in New York City	2094 (2012-2018)	White, Black, Hispanic, Asian, and other race	Time to treatment	Racial or ethnic minority groups had consistently longer intervals to treatment, with Black women experiencing the greatest disparity. Time from initial comprehensive breast center visit to treatment was also significantly shorter in White patients vs non-White patients.
Sukniam et al, 2022^31^	To identify the demographic/socioeconomic factors associated with disparities in time to breast cancer treatment	Retrospective/NCDB	715 210 (2008-2019)	White, Black, Native American, Asian, other race, non-Hispanic, Hispanic/time to surgery, chemotherapy, radiotherapy	Time to first treatment, time to surgery, time to chemotherapy, and time to first radiation.	Hispanic patients had the longest times to surgery, radiation, and chemotherapy compared with non-Hispanic patients. Black patients, those who were uninsured, and those with lower income had the longest times to treatment.
Taparra et al, 2022^60^	To assess disparities among women who self-identify as Asian American with respect to overall survival and surgery-to-radiation intervals	Retrospective cohort study/NCDB	578 927 (2004-2017)	NHW, East Asian, South Asian, Southeast Asian, Native Hawaiian, Micronesian, Chamorra, Guamanian, Polynesian, Tahitian, S amoan, Ton-gan, Melanesian, Fiji Islander, New Guinean, and other Pacific Islander/surgery-to-radiation intervals	Overall survival and time to surgery	Hazard ratio was significantly different across racial groups. Surgery-to-radiation intervals was statistically significant within some racial groups.

**Table 2:** Continued

**Reference**	**Study objective(s)**	**Study design and source of data**	**Sample size (data collection period)**	**Race categories, time to treatment(s)**	**Outcomes**	**Results or conclusion**
Tjoe et al, 2022^61^	To determine factors associated with a delay in time to surgery and disease-free survival	A retrospective case-control study/a community-based 15-hospital health system	4462 (2006-2016)	Race: NHW, NHB, Hispanic, Asian, Pacific Islander, other race/time to surgery	Time to surgery	Significant differences were found across racial groups.
Verdone et al, 2022^62^	To provide a tool to predict patient socioeconomic factors associated with risk for delay	Retrospective cohort study/NCDB	514 187 (2004-2017)	Race: White, Black, American Indian, Aleutian, Eskimo, Asian, and Hawaiian, Pacific Islander. Ethnicity: non-Spanish, non-Hispanic, Spanish, Hispanic/time to surgery from diagnosis	Time to surgery	The estimated number of days from diagnosis to surgery was significantly longer for all racial groups compared with White patients.
Chen et al, 2023^63^	To evaluate time to surgery by race	Retrospective cohort study/NCDB	866 840 (2010-2019)	Race: White and Black. Time to surgery	Time to surgery	The odds of surgical treatment in Black patients was significantly lower than White patients.
Patel et al, 2023^64^	To identify differences in time to treatment in Asian and Pacific Islander patients	Retrospective cohort study/NCDB	1 670 528 (2004-2017)	Race: White, Chinese, Japanese, Filipino, Native Hawaiian, Korean, Vietnamese, Laotian, Hmong, Kampuchean, Thai, Asian Indian and Pakistani, Pacific Islander Time to surgery	Time to surgery	Race was associated with time to surgery.
Chavez-MacGregor et al, 2023^65^	To examine the association between Medicaid expansion and adjuvant chemotherapy initiation delays according to race and ethnicity	Retrospective cohort study/NCDB	100 643 (2007-2017)	Race/ethnicity: American Indian and Alaska Native non-Hispanic, Asian American non-Hispanic, Black, Hispanic, Pacific Islander non-Hispanic, other or unknown race, White/time from surgery to chemotherapy	Time to adjuvant chemotherapy	Statistically significant reductions in time to chemotherapy were observed among White patients and those belonging to racialized groups.
Beaulieu-Jones et al, 2024^32^	Performance of safety-net hospitals in delivering timely care for all patients	Retrospective cohort study/an institutional tumor registry	799 (2009-2019)	Race (Black and White), ethnicity (Hispanic and non-Hispanic). Time from tissue diagnosis to initial treatment	Time to treatment and occurrence of treatment delay	No significant effect of race or ethnicity on time to treatment was observed.

Abbreviations: CCR, California Cancer Registry; MHS, Military Health Service; NCDB, National Cancer Database; NHB, non-Hispanic Black; NHW, non-Hispanic White; SES, socioeconomic status.

An overview of sample sizes and study periods specified by databases in the United States is summarized in the Supplementary Materials. The results demonstrate the predominant use of administrative data from the National Cancer Database in the studies included in this review (Figure S41).

### Time to treatment initiation

**Figure 2:**
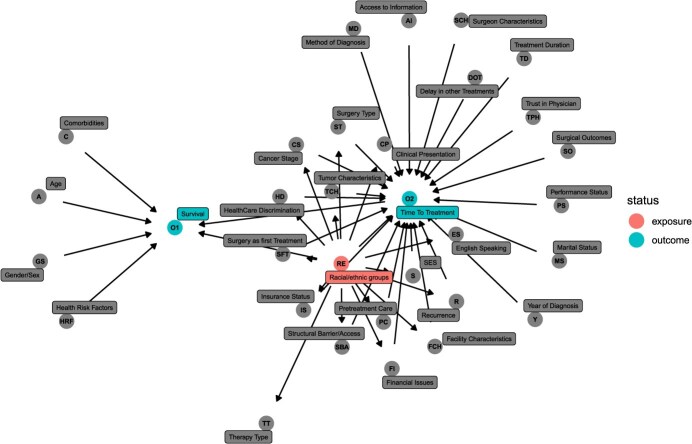
A composite causal directed acyclic graph: factors affecting time to breast cancer treatments and survival across racial/ethnic groups, as conceptualized in current literature.

The included studies used various measures of treatment initiation.^21^^,^^27–50^^,^^52^^,^^53^^,^^55–65^ These included TTI (ie, first treatment), time to treatment completion, and specific treatment of interest, such as surgery and chemotherapy. Figure 2 shows the comprehensive causal DAG of the factors examined by these studies on the implied causal pathways to time to treatment and survival across different racial/ethnic groups.

#### Delay in time to first treatment

Six studies focused on the time to initiation of any type of treatment in patients with breast cancer.^27‑34^ Studies on time to first treatment found higher risk of delay in Black, Hispanic, or other non-White races or ethnicities compared with White patients.^27‑29^ One study on delays in treatment initiation and treatment duration found that delayed initiation among Black patients was not significantly associated with either SES or access to care; however, low SES and more barriers were linked to longer treatment duration across Black and White groups.^28^

#### Delay in time to surgery

Time to surgery was specifically assessed in many studies, with most considering a wait of longer than 1 month as a delay in initiation of assigned surgery.^34–49^^,^^52^^,^^53^^,^^55^^,^^56^^,^^58–64^ Mean time to definitive breast cancer surgery was significantly longer for Black patients than White patients.^36^^,^^43^ Comparing factors across non-Hispanic Black and non-Hispanic White groups showed that Black patients treated at high-volume hospitals had higher rates of surgical delay but were less likely to undergo low-value surgical procedures compared with White patients.^55^ A comparative analysis of this study showed significant disparities in the time to surgical treatment for breast cancer among Hispanic, non-Hispanic Black, and minority Asian ethnic subgroups compared with non-Hispanic White patients.^55^ One study suggested that treatment delays among Black, Hispanic, and other non-White patients were explained mostly by disparities in education, comorbid conditions, insurance, and facility type.^45^ Another study found the timeliness of care was not influenced by patient race; however, Hispanic patients were significantly less likely to undergo surgery within 1 month.^48^^,^^56^ Although immediate breast reconstruction delays time to definitive surgery, its use did not substantially affect time to adjuvant treatment or survival outcomes.^48^

#### Delay in time to chemotherapy

Studies addressed factors affecting the timing of chemotherapy in patients who are candidates for surgery.^48^^,^^52^^,^^53^^,^^63^^,^^65^ Although no significant difference was found across racial/ethnic groups, mastectomy with immediate reconstruction in candidates for breast conservation independently predicted delay in initiation of adjuvant chemotherapy.^48^ One study indicated a significant difference in the delay of chemotherapy initiation across racial/ethnic groups, with adverse outcomes linked to a delay in initiating adjuvant chemotherapy of 3 months or longer.^52^

### Ethnoracial disparities, time to treatment, and survival

Seven studies addressed both time to treatment and survival together.^21^^,^^37^^,^^39^^,^^42^^,^^52^^,^^57^^,^^60^ A large retrospective study that assessed disparities between Asian Americans with Native Hawaiians and Other Pacific Islanders (NHPIs) found NHPIs had worse survival compared with non-Hispanic White patients, whereas all Asian American subpopulations had improved survival, with Southeast Asians and NHPIs experiencing longer times to treatment initiation.^60^ One study found that longer time to surgery correlated with poorer overall survival in breast cancer across different ethnoracial categories, after adjusting for time to treatment.^42^

### Comprehensive summary of causal pathways

The comprehensive DAG in Figure 2 includes all assessed factors affecting time to treatment and survival in patients with breast cancer and their relationships, providing a visual summary of the causal conceptualization in the summarized literature (see R code in Appendix S2). Note that although the comprehensive DAG shows many mediated pathways, these were often constructed of implied pathways from multiple studies that included portions of the pathway.

### Identified unmeasured factors affecting TTI and survival

Most included studies used administrative records of cancer registry programs, with only 1 undertaking primary data collection. Given limitations of secondary data sets, authors highlighted numerous unmeasured factors that could significantly affect the initiation of treatment in patients with breast cancer; these are summarized in Table 3. These factors change over time; for instance, an earlier study identified additional imaging or biopsy procedures as key missing factors affecting the time to treatment, and subsequent research showed that these factors exhibit variations across different SES levels, and ultimately these were associated with racial/ethnic groups.^11^^,^^12^

**Table 3:** List of unmeasured factors specified by authors in include studies.

**Reference**	**Unmeasured factors**
Hershman et al, 2005^21^	Performance status, socioeconomic status, and obesity
Alderman et al, 2010^34^	Surgical complications, survival/recurrence, low-volume vs high-volume clinic setting
Chandwani et al, 2014^35^	Additional test to investigate preoperative magnetic resonance imaging
Sheppard et al, 2015^36^	Stage migration of disease
Buckley et al, 2017^38^	Type of reconstruction
Jabo et al, 2018^39^	Social support, not seeking care as instructed, comorbidity, human epidermal growth factor receptor 2 status, insurance barrier or operating room availability, physician driven such as overbooked clinics, preoperative imaging/testing
Larson et al, 2018^51^	Zip code, type of hospital, specific chemotherapy
Eaglehouse et al, 2019^42^	Erythroblastic oncogene B status
Kupstas et al, 2019^52^	Specific chemotherapy regimen, length of treatment, completion of chemotherapy, recurrence
Emerson et al, 2020^28^	Distance to care, type of care center, workload associated with treatment. Specific financial and transportation issues, biological factors, facility information
Prakash et al, 2021^53^	Disease-specific survival, recurrence
Jackson et al, 2021^55^	Genetic information
Dankwa-Mullan et al, 2021^56^	No pathology variables, patients preferred surgery type, no data on health insurance
Blazek et al, 2021^57^	Hormone receptor status
Chagpar et al, 2022^58^	Patient comorbid conditions, insurance status, education, income, insurance, and health literacy
Navarro et al, 2022^59^	Family history of breast cancer, breast cancer type, patient’s nativity and immigration status
Schermerhorn et al, 2022^29^	Individual-level income, individual-level education, individual experience of discrimination
Sukniam et al, 2022^31^	Quality of life, recurrence-free survival, progression-free survival
Chen et al, 2023^63^	Patient-level socioeconomic status, individual level income, additional imaging, and multiple provider consultation
Beaulieu-Jones et al, 2024^32^	Housing insecurity, comorbid mental health conditions, food insecurity, access to transportation, and proximity to the health system, barriers to breast cancer screening and/or diagnosis

## Discussion

Our primary goal for this review was to explore the current evidence for ethnoracial disparities in breast cancer survival. There is compelling evidence indicating ethnoracial disparities in TTI and survival among patients with breast cancer.^66–68^

Racial disparities have been assessed by examining various set of factors, including demographics and tumor characteristics, and confounders that contribute to these disparities.^28–31^^,^^35^^,^^41^^,^^42^^,^^45^^,^^58^^,^^59^^,^^67^^,^^69^ Studies found time to surgery varied notably between private and public settings, with both system-based issues and patient sociodemographic factors (eg, race, marital status, insurance) posing barriers to timely care.^69^ These variations underscore the complexity of defining and measuring treatment delays in breast cancer care. Based on the literature, many factors have been assessed, and studies suggested that equity-focused interventions are needed to address the disparities to improve patients’ survival.^67^

These disparities in the timing of breast cancer treatment initiation and completion are clear, with delays significantly impacting patients’ survival.^29^^,^^35^^,^^42^^,^^45^^,^^59^^,^^60^ Studies consistently report that Black patients face longer wait times before undergoing definitive surgery for breast cancer.^36^^,^^43^ At the individual patient level, these disparities highlight the importance of improving surgeon-patient communication, decision-making, and care coordination throughout neoadjuvant systemic therapy and the perioperative period.^39^ At the system level, they highlight the need for a nuanced understanding of the driving factors of delays and their causal relationships (as expressed in the DAGs) and structural barriers to ensure that system-level interventions to reduce delays are effective.

### Focus on individual versus structural factors

Although studies frequently identified unmeasured factors that were deemed important, (Table 3), there was an exclusive focus on individual-level rather than structural factors that lead to delays in treatment and poorer survival. For example, studies did not measure or examine the relationship between structural racism, structural sexism, and their intersections. Neither did studies examine the interaction between health system–level factors, such as quality of care, and individual-level factors such as SES. This lack of consideration for broader structural influences limits our understanding of the complex interplay among tumor, societal, health care system, and individual-level factors in shaping breast cancer outcomes. One large cohort study that specifically discussed system-level factors (ie, hospital level) found significant differences in time to surgery for Black women compared with White women among patients with nonmetastatic invasive breast cancer.^63^ This difference varied across different hospitals and regions in the country, suggesting that hospital factors play an important role in disparities in surgical care. However, studied system-level properties were considered as individual-level factors in the analysis.^63^

The lack of a system-level lens in this area led us to consider how disparity has been conceptualized in this field and to consider what drivers of disparity, both individual and structural, may be missing from this conceptualization. The existence of a substantial number of factors, despite not being included in the earlier survey research, demonstrates a significant gap in the body of knowledge regarding disparities in breast cancer treatment and survival. This observation underscores the predominant focus of research on breast cancer at the individual level. Although individual factors undoubtedly play a significant role in shaping breast cancer outcomes, the emphasis on higher-level systemic and societal influences is essential to comprehensively understand the complexities of this disease.^70^ By recognizing the broader structural determinants that affect breast cancer incidence, treatment access, and outcomes, researchers can develop more holistic approaches that address the full spectrum of factors contributing to ethnoracial disparities in breast cancer.

### Overlooked determinants in breast cancer treatment disparities

In this review, we identified unmeasured factors from the included studies that were not captured in the final analyses, as detailed in Table 3. If these factors act as confounders, they must be addressed because their effects might obscure the true extent of racial disparities in breast cancer. It is important to note that despite our extensive search, there remain additional factors not covered by the studies included in this review, indicating ongoing gaps in the literature to consider for future intervention.

In constructing our DAGs, we incorporated structural, systemic factors such as socioeconomic conditions and community-level resources, as well as individual, immediate influences, such as access to health care and personal health behaviors, to better capture the contextual and systemic determinants of racial health disparities in breast cancer. Addressing gaps in the current literature, some interventions outside our included studies provide examples of effective strategies. For instance, a tracking and feedback registry aimed at improving continuity of care among Black and Hispanic women with early-stage breast cancer led to notable improvements, including increased number of oncology consultations and reduced underuse of adjuvant treatments, effectively eliminating racial disparities in treatment utilization. This registry intervention improved coordination between surgeons and oncologists, illustrating how systemic changes can enhance treatment adherence and promote equity in cancer care.^71^ Recognizing and addressing these gaps could lead to more effective strategies for reducing treatment disparities among marginalized populations.

### Causal interpretation of race/ethnicity

The causal interpretation of race/ethnicity is challenging.^72^ Studies mainly investigated the association of TTI and survival, controlling for confounders such as age and stage, across racial/ethnic groups. This may be because many factors that affect ethnoracial inequities in health are rarely measured in breast cancer studies and do not typically exist in registry data; for example, racist experiences in health systems, patients’ mental health or quality of life, and individual-level socioeconomics.^31^^,^^32^ The persistence of not measuring important causal factors and not attending to causal considerations within the context of ethnoracial health disparities in breast cancer remains a concern, because their absence impedes our ability to develop and assess interventions designed to redress such disparities. Our findings align with recent critiques that emphasize the need for comprehensive and context-specific approaches in causal inference.^19^ Although our DAGs accounted for key confounders, limitations related to unmeasured contextual factors highlight the importance of future research in refining these models.

However, the existing literature on racial health disparities primarily focuses on differences in outcomes rather than examining the specific factors or path-specific effects that contribute to health inequities. Moreover, there is a notable gap in understanding the causal mechanisms underlying these disparities, not only in breast cancer but also in broader studies of racial health disparities. Causal inference literature has introduced the causal decomposition method,^72^^,^^73^ which could be particularly effective in exploring racial disparities in breast cancer. Incorporating this approach into analysis of racial disparities in breast cancer would underscore the importance of using equity-focused methodologies.^74^ By using these advanced causal techniques, researchers can better understand and address the specific mechanisms driving racial disparities in breast cancer outcomes.

### Two decades of research on racial and ethnic differences

Furthermore, the studies included in this review span more than 2 decades, a period marked by important advancements in breast cancer treatment, methodological innovation, and growing awareness of racial and ethnic health disparities. Although a formal temporal analysis was beyond the scope of this review, it is likely that changes in clinical standards, data infrastructure, and conceptual frameworks have shaped both the design and interpretation of studies over time. For instance, more recent research may use sophisticated analytical strategies, draw on larger and more diverse data sets, and more explicitly engage with concepts such as structural racism or intersectionality.^29^^,^^64^^,^^65^ Although the studies included in our review consistently reported disparities in timely treatment and survival across racial/ethnic groups, no clear trends were observed in how the magnitude or framing of these disparities changed over time. These evolving contexts highlight the need for research to systematically explore how temporal shifts influence the detection and interpretation of disparities in breast cancer outcomes.

### Limitations

We only set out to retrieve documents in English and, given our exclusively US-based results, our search strategy may have inadvertently excluded work in other jurisdictions where the discourse on ethnoracial disparity is framed differently. We also excluded gray literature, which may have excluded community and popular perceptions of such disparities in breast cancer.

Additionally, the potential for unmeasured confounders remains a limitation in studies using DAGs. The absence of causal designs in studies, for example, using matching, propensity scores, identification, and control of confounders, as well as the lack of mediation models, limit our ability to assess with certainty what the causal assumptions of the authors might have been. Although some studies implied the presence of mediation models, there was a lack of formal mediation analysis in this context. Future research should aim to integrate more comprehensive data to address this issue.

Although our focus on the United States is acknowledged as a limitation, it is also important to note that the measurement and assessment of race/ethnicity in the United States are unique compared with most other parts of the world. This specificity may influence how race- and ethnicity-related factors are understood and could serve as effect modifiers of the DAGs identified in this study. The US focus, therefore, is not necessarily a negative but rather an aspect that requires careful consideration when generalizing findings to other geographic settings.

Finally, a potential limitation of this review is the absence of a formal assessment of the impact of calendar year on study findings. Given the broad 25-year time frame encompassed by the included studies, temporal changes in treatment protocols, data quality, and approaches to measuring and conceptualizing disparities may have contributed to heterogeneity in the reported outcomes. Future reviews and meta-analyses would benefit from explicitly examining temporal trends to better understand how shifts in research and clinical practice influence the characterization of racial and ethnic disparities in breast cancer care and outcomes.

### Conclusion

There are persistent disparities observed in treatment and mortality outcomes among different racial/ethnic groups, particularly between White and Black female patients with breast cancer. This highlights the importance of addressing and eliminating ethnoracial disparities in breast cancer care to ensure equitable access to timely and effective treatments for all patients, ultimately improving long-term survival rates across diverse populations. Our study underscores the need for context-specific causal frameworks to address racial disparities in breast cancer treatment, contributing to the growing body of research advocating for methodological rigor in causal inference. Future research must adopt a more inclusive and multilevel approach, incorporating systemic and societal factors alongside individual-level determinants to advance our understanding and improve interventions in breast cancer care.

## Supplementary Material

Web_Material_mxaf009

## Data Availability

All data used in this systematic review are from published studies, which are referenced in the article. No new data were generated or analyzed in this study.
