# Supplementary material for: Ethnoracial disparities in breast cancer treatment time and survival: a systematic review with a DAG–based causal model
Source: Epidemiol Rev. 2025 Jun 6;47(1):mxaf009. doi: 10.1093/epirev/mxaf009 (PMC12278384; doi:10.1093/epirev/mxaf009)
Supplement: Web_Material_mxaf009 [file web_material_mxaf009.zip › Supplement.docx]

**Ethnoracial Disparities in Breast Cancer Treatment Time and Survival: A Systematic Review With a DAG-based Causal Model**

Parisa M. Hesari, Drexler James, Daniel J. Lizotte, Greta R. Bauer

**Supplementary Materials**

**Appendix S1**

Table S1: Search strategy used in all databases

Figures S1–S40: Study-specific Directed Acyclic Graphs (DAGs) and R code for each included study

Figure S41: Sample sizes and study time periods of different databases in included studies

**Appendix S2**: R code for the finalized summary DAG

Table S1, a search string of all databases

| All databases will be searched using the combination of keywords ‘breast cancer’ and ‘time to treatment’ with their MeSH terms. As an example, search strings in PubMed were as follows: (((((((((((breast cancer[MeSH Terms]) AND (“time to treatment”[Title/Abstract]) OR (“time to surgery”[Title/Abstract]) OR (“time to chemotherapy”[Title/Abstract]) OR (“time to radiotherapy”[Title/Abstract]) OR (“time to hormone therapy”[Title/Abstract]) OR (“time to endocrine therapy”[Title/Abstract]) OR (“delayed treatment”[Title/Abstract] OR (“delayed treatments”[Title/Abstract])). |
| --- |

Figures S1–S40: Study-specific Directed Acyclic Graphs (DAGs) and R code for each included study

*#Load required Packages***library**(dagitty)
**library**(ggdag)

**library**(ggplot2)

**set.seed**(2024) *#1:eFig1, Hershman et al, 2005 [19] -----*dagRace<-**dagify**(TTT**~**Race**+** ethnicity**+**Age**+** Stage**+** EnglishSpeaking**+**HR**+**Grade**+** Comorbidities**+** ChemoDoxo**+**TreatmentCom**+**Delay,
 TreatmentCom**~**Race,
 Stage**~**Race,
 Grade**~**Race,
 Delay**~**Race,
 exposure="Race",
 outcome="TTT")
dag_plot1 <- **ggdag_adjust**(dagRace,
 var = "TTT",
 node_size = 16,
 text_size = 2,
 label_size = text_size,
 text_col = "black",
 label_col = text_col,
 node = TRUE,
 stylized = FALSE,
 text = TRUE,
 use_labels = NULL,
 collider_lines = FALSE
) **+**
 **theme_void**()

**print**(dag_plot1)

_
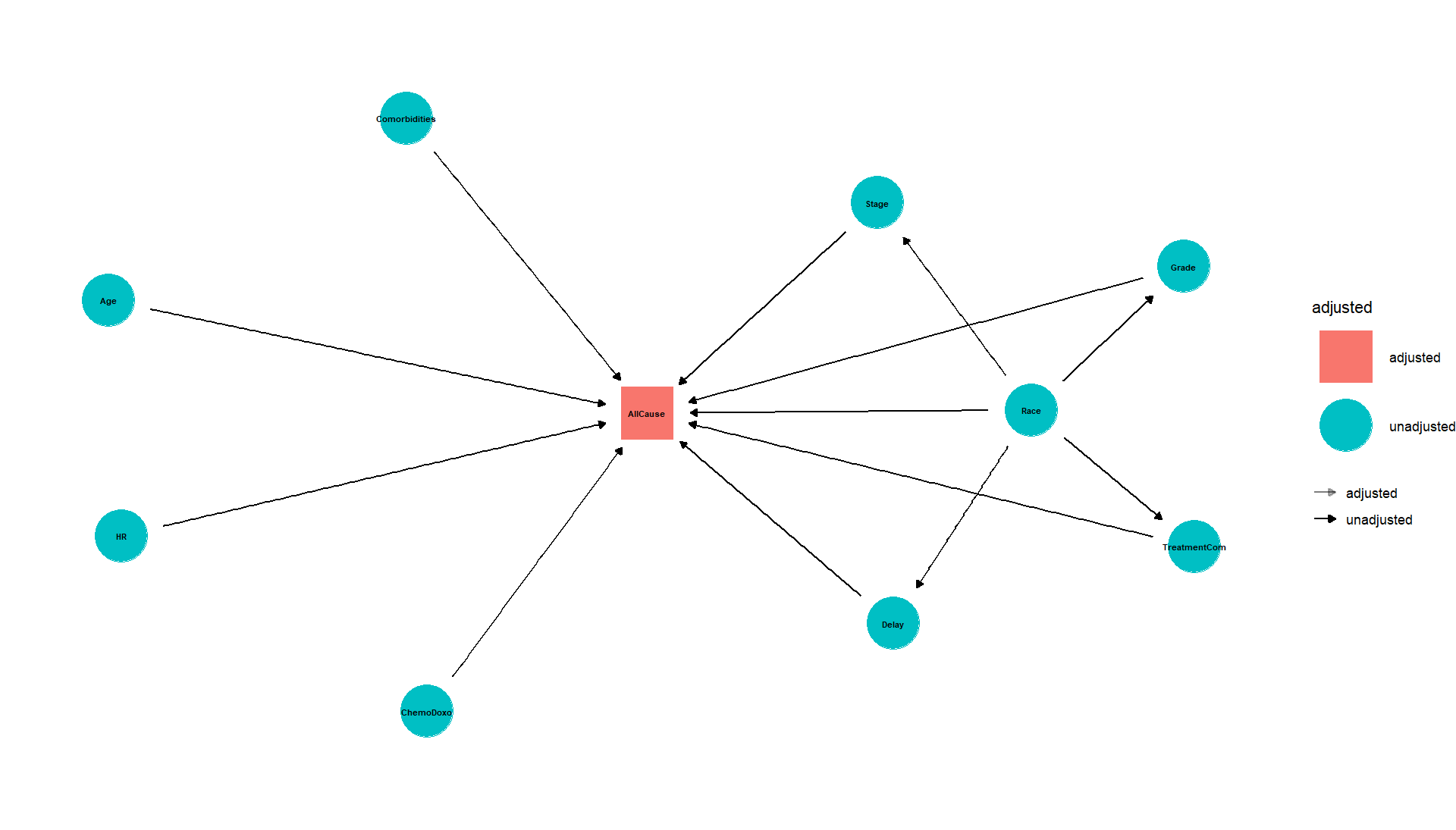
_

*#2:eFig2, Alderman et al, 2010 [35]*


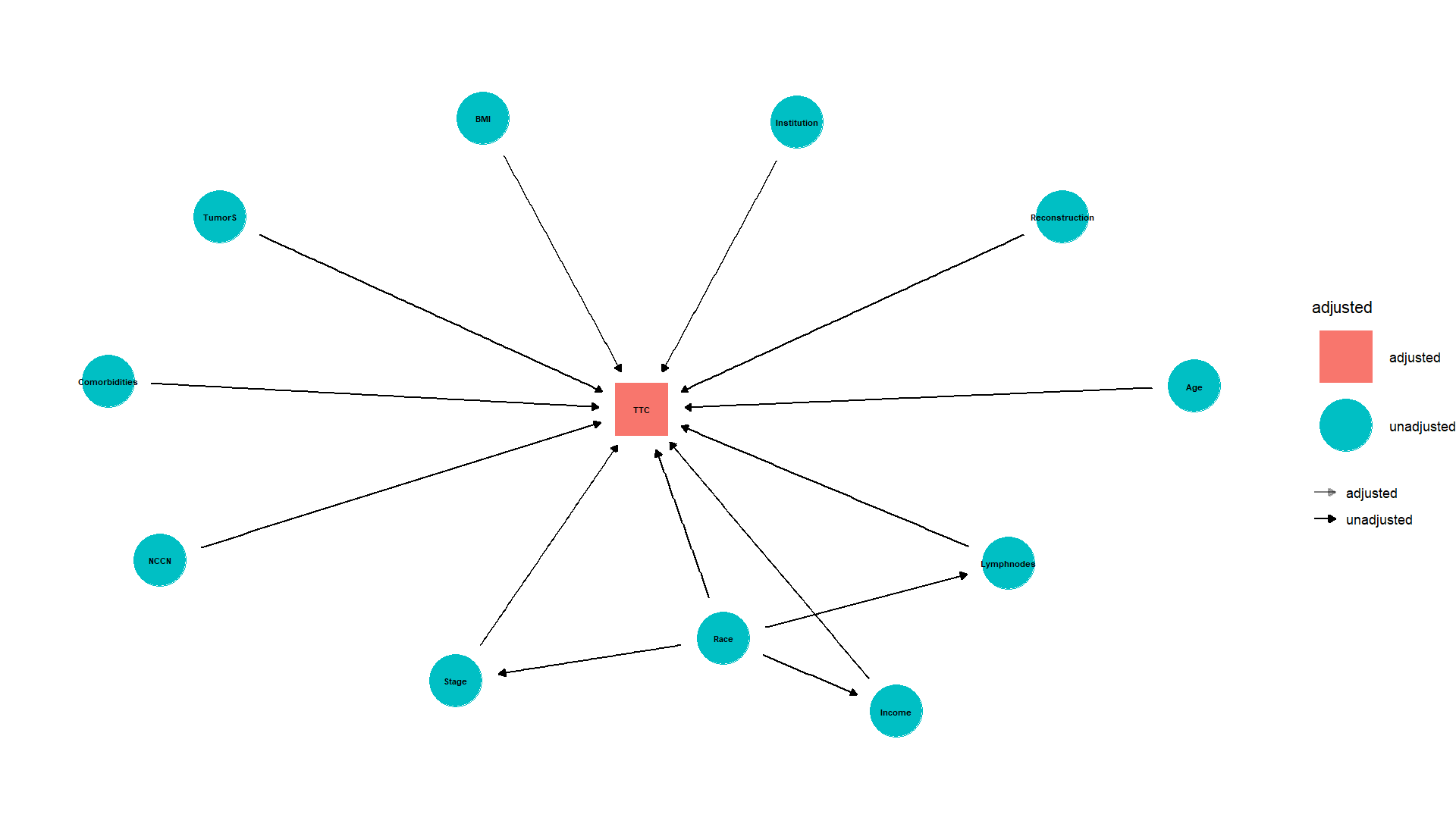


*#3:eFig3, Fedewa et al, 2011 [28]*


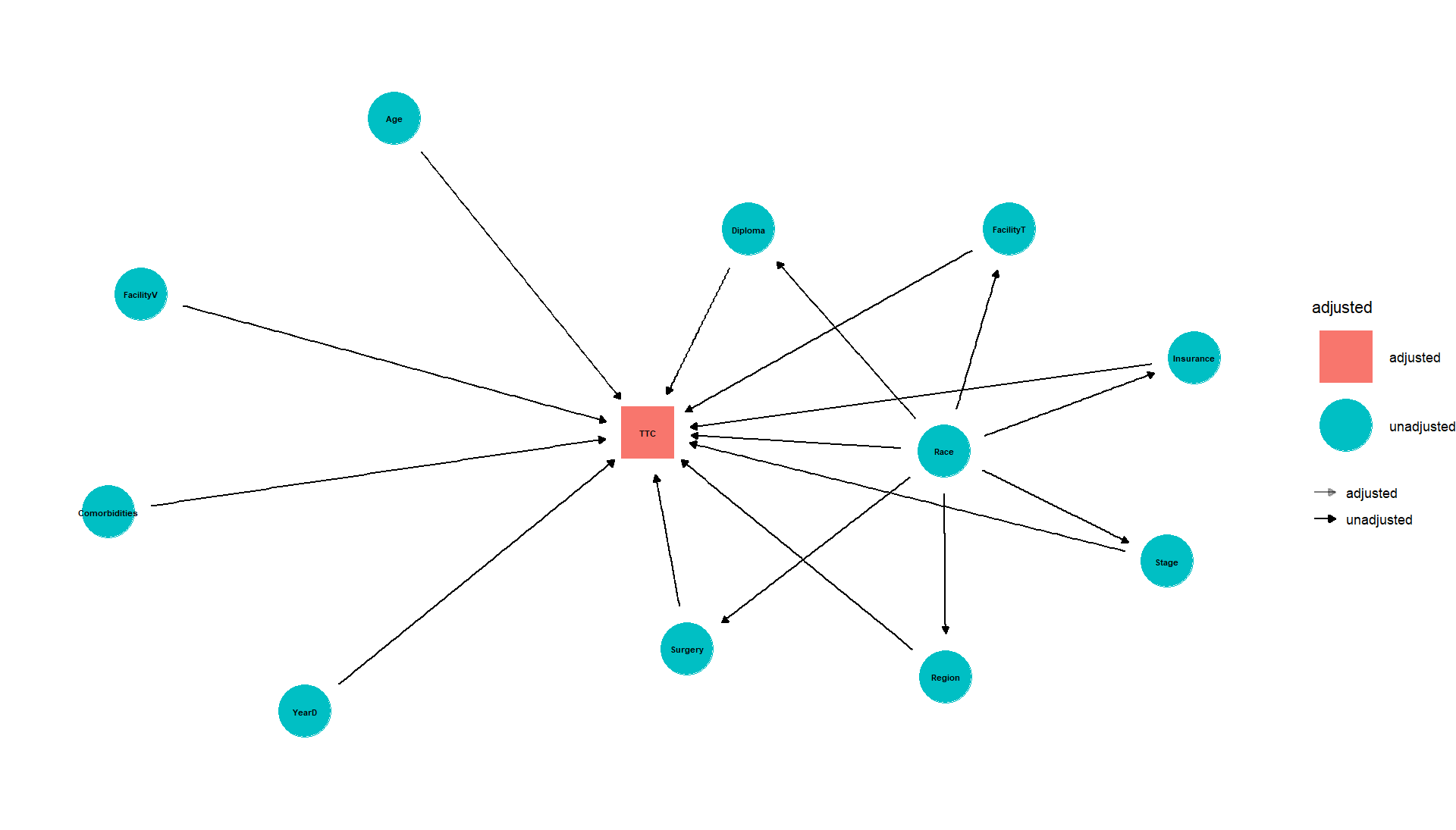


*#4:eFig4, Bradly et al 2012[59]*


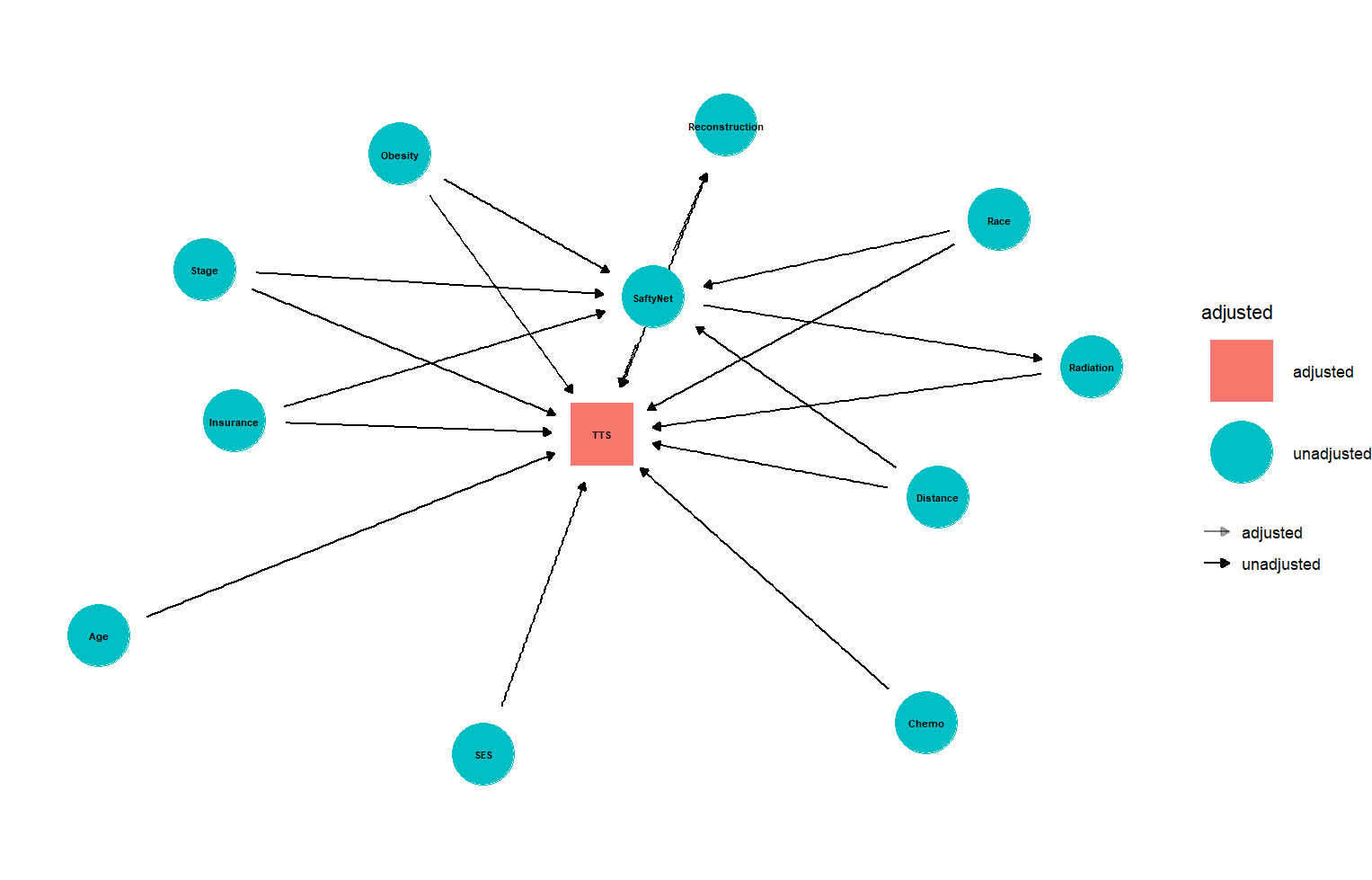


*#5:eFig5, Mosunjac et al, 2012[60]*


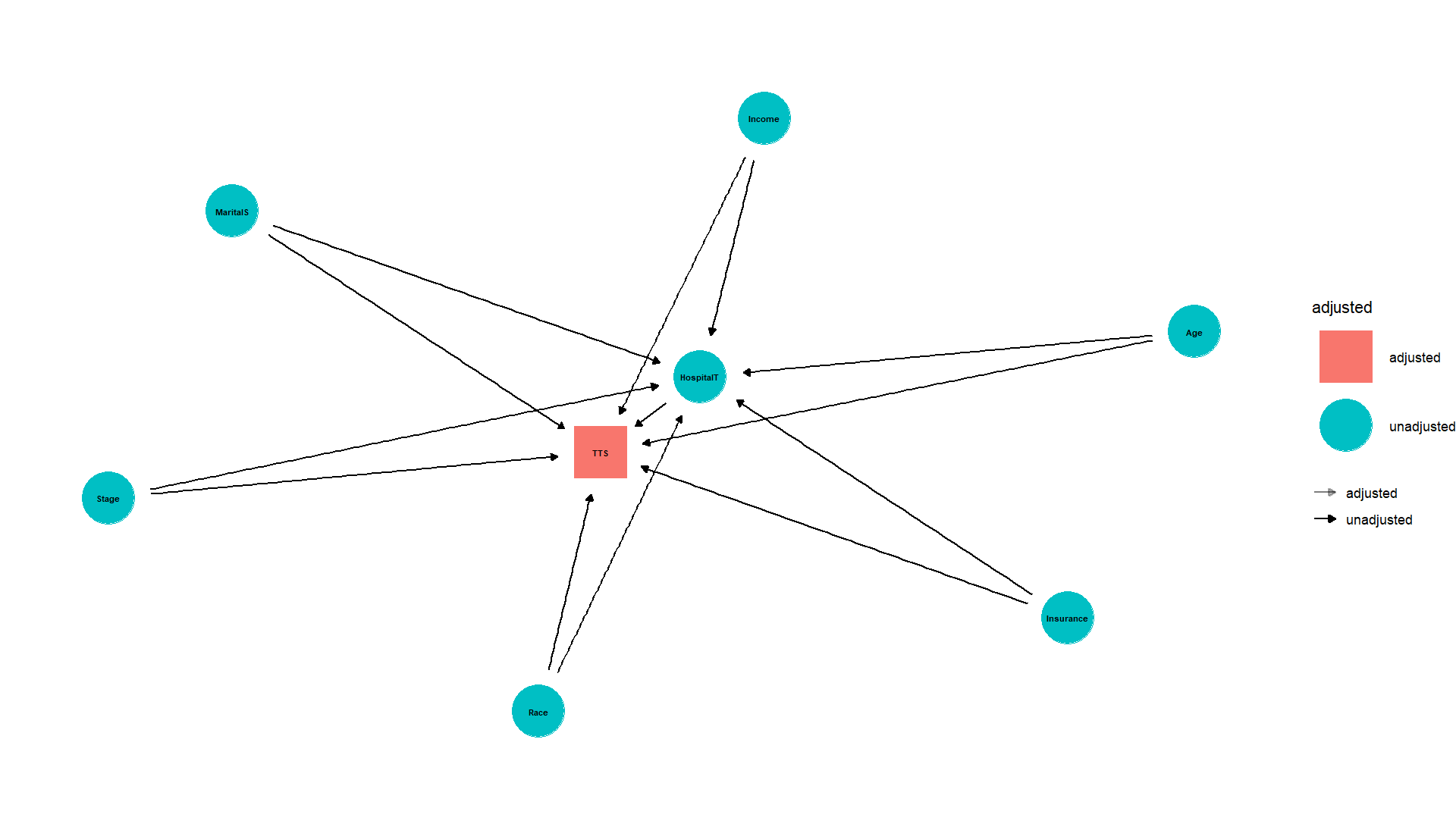


*#6:eFig6, Barry rt al, 2014 [55]*


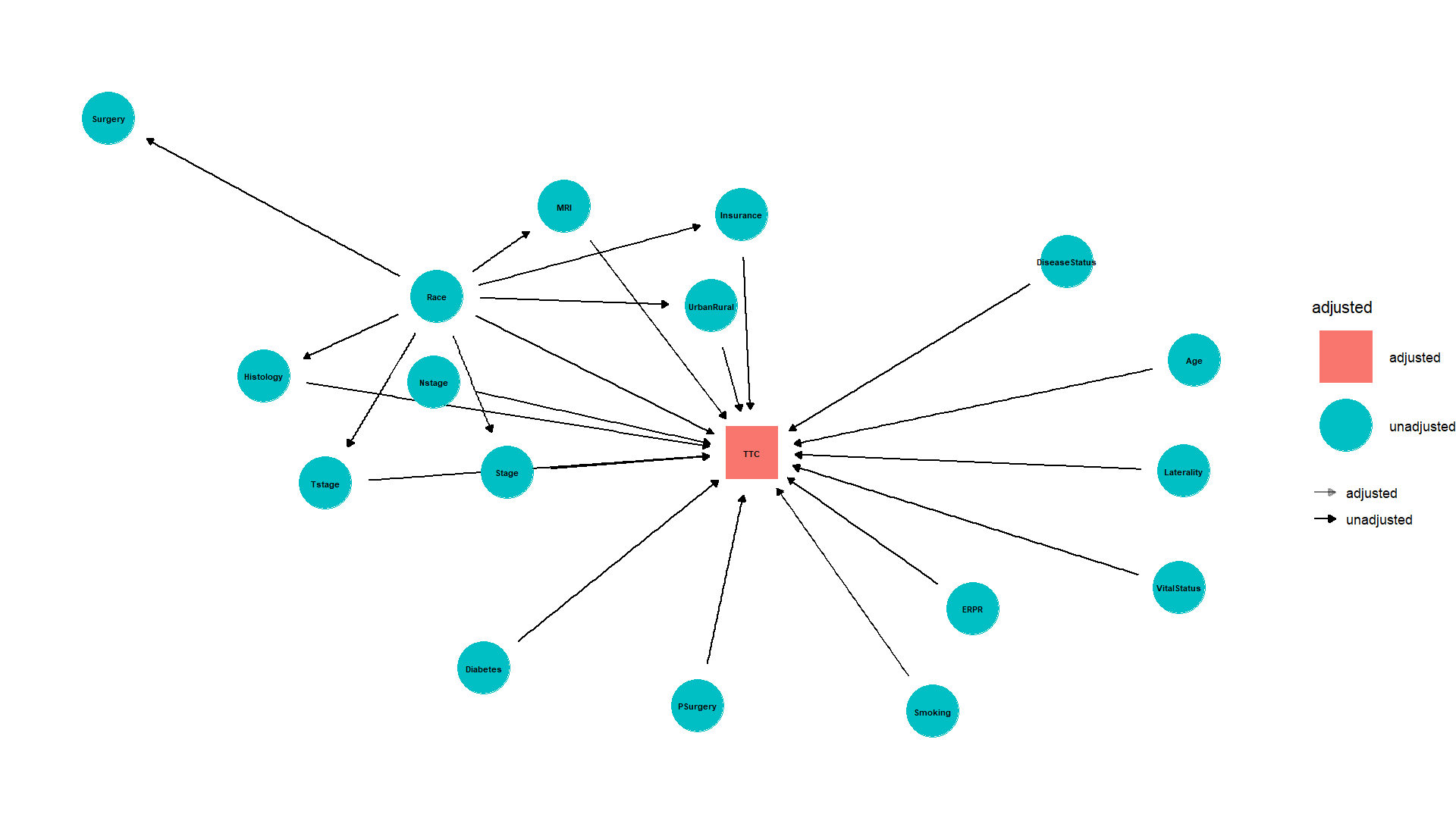


*#7:eFig7, Liederbach et al 2014[61]*


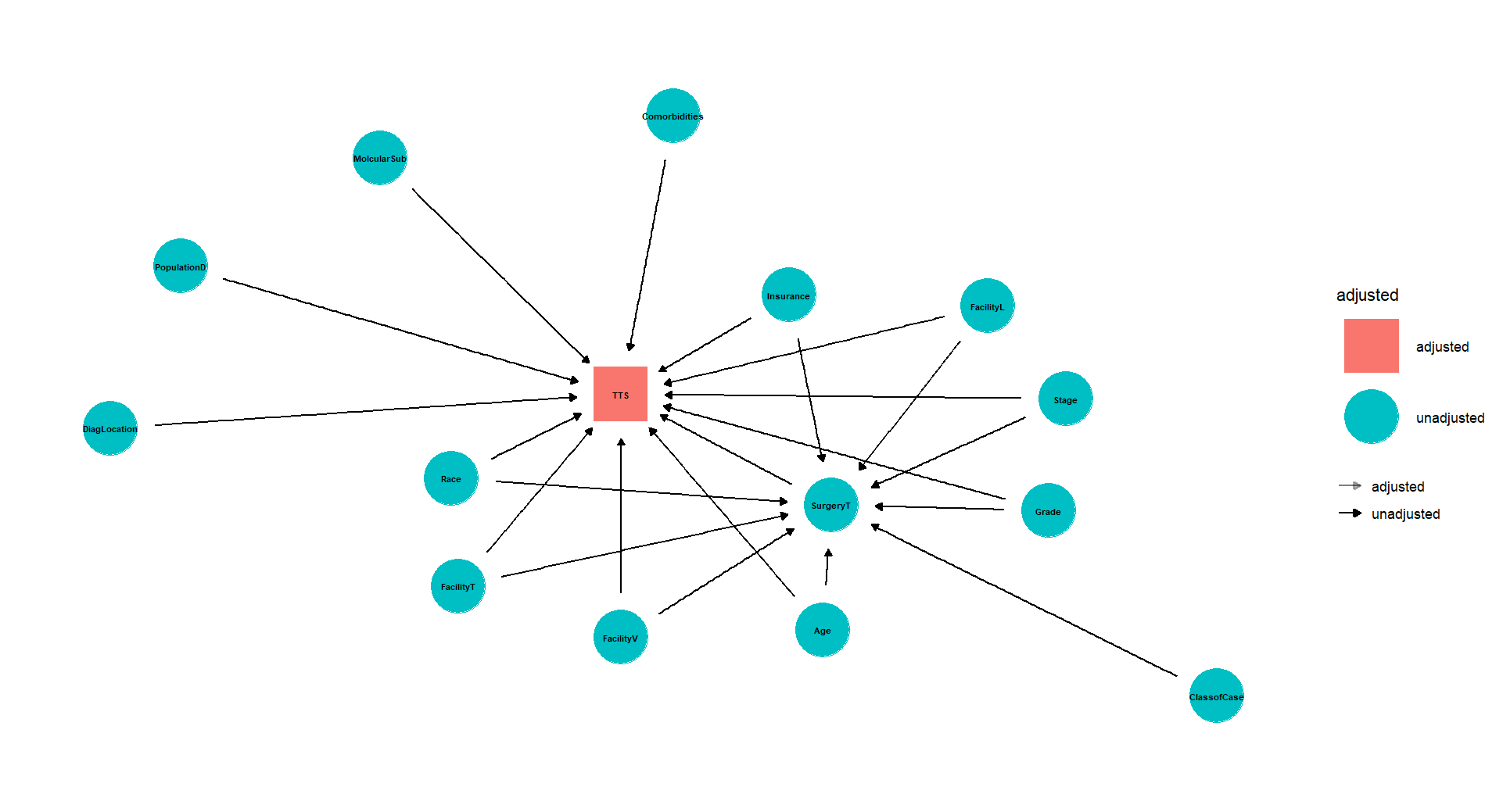


*#8:eFig8, Chandwani et al, 2014 [36]*


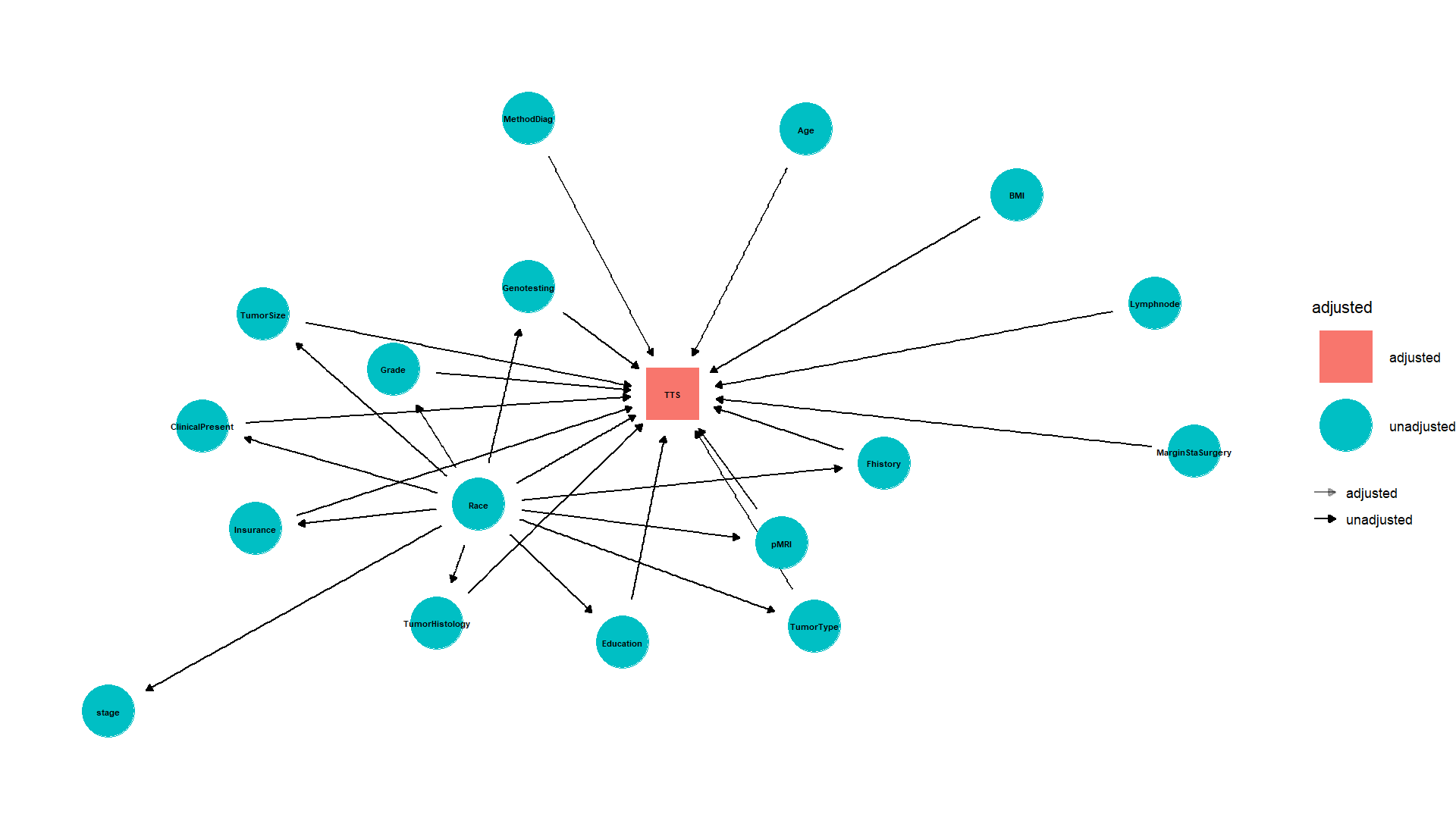


*#9: eFig9, Sheppared et al 2015 [37]*


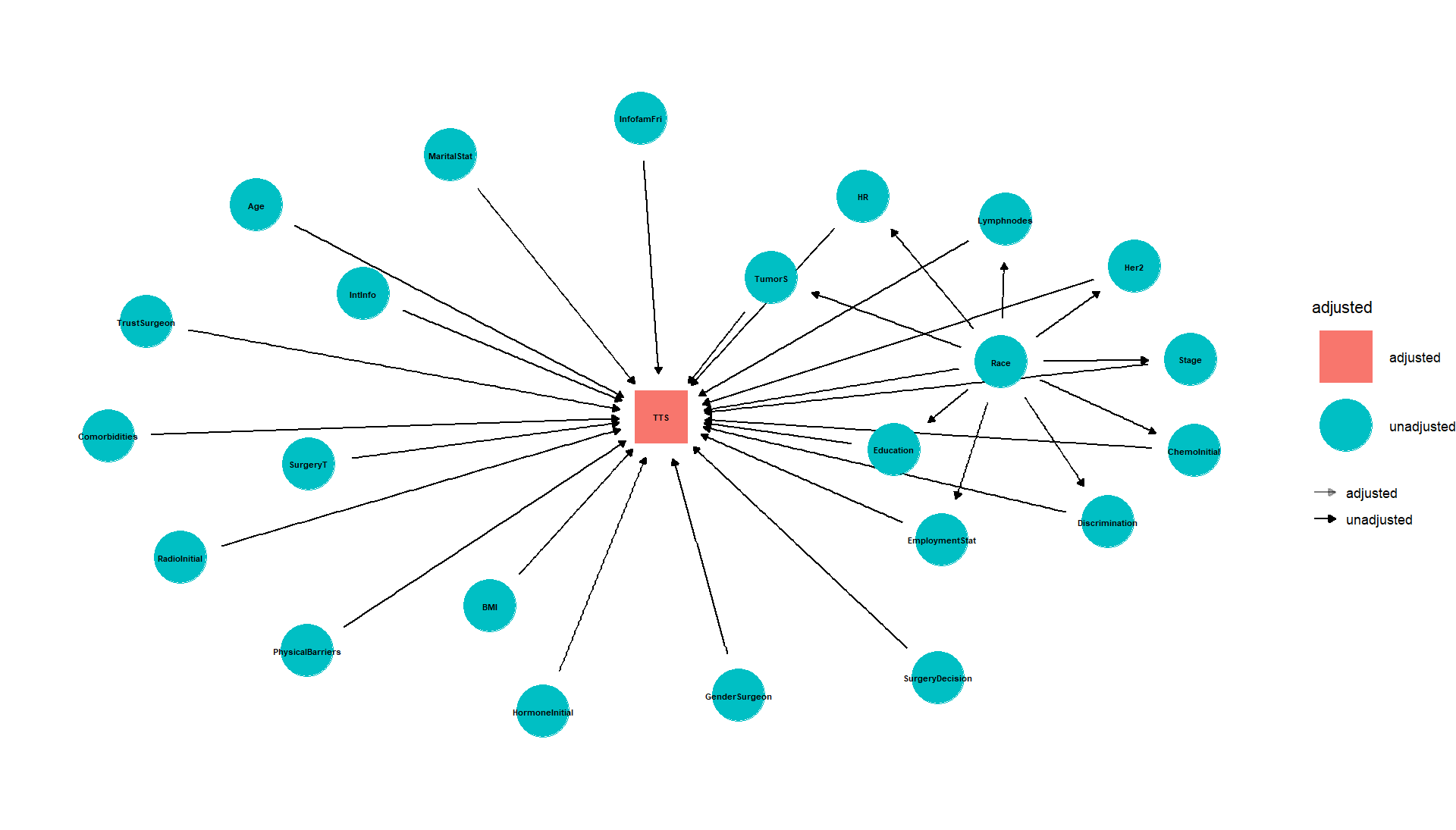


*#10:eFig10, Chavez-MacGregor et al, 2016 [63]*


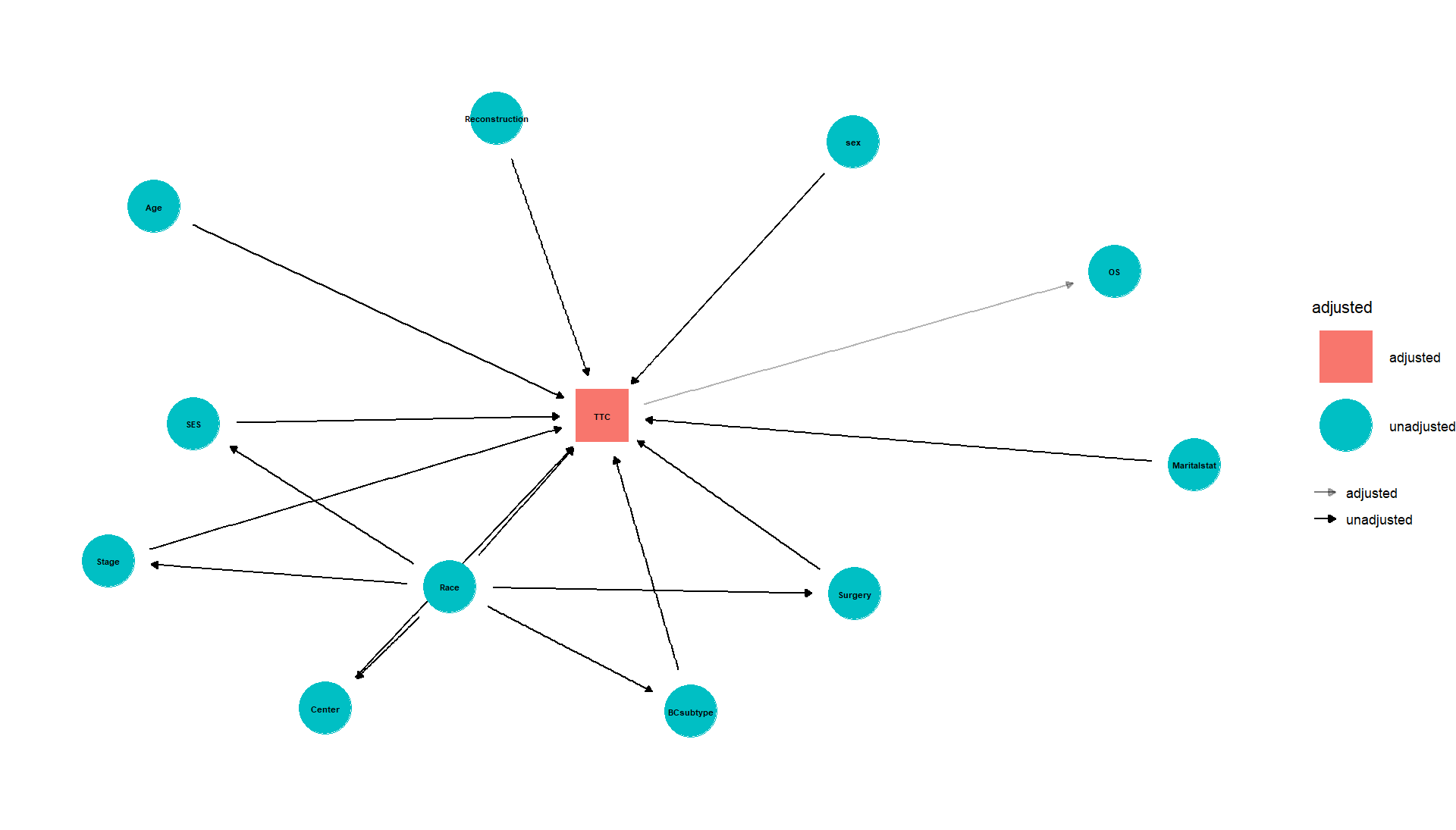


*#11:eFig11, Sanford et al, 2016[38]*


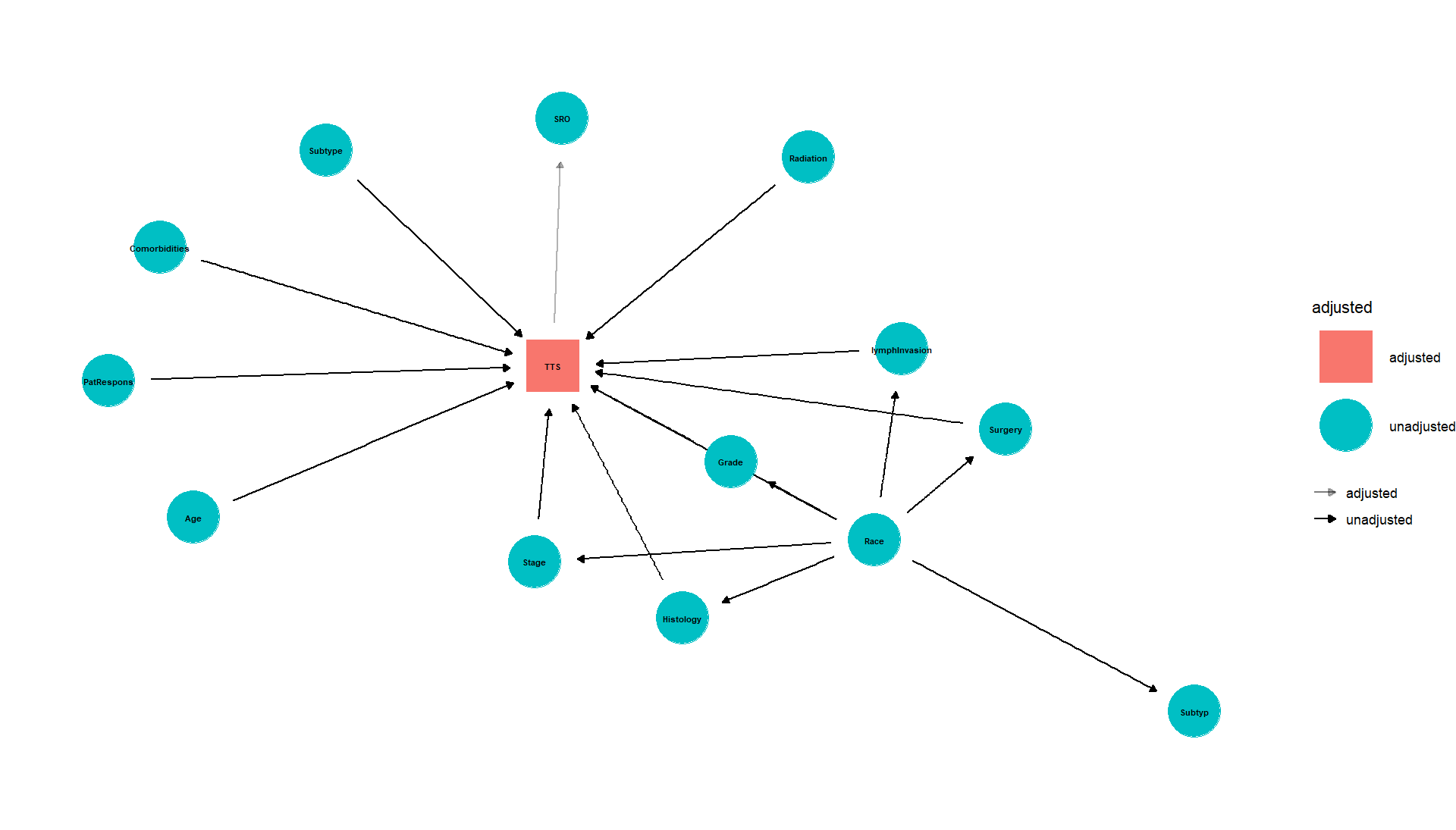


*#12:eFig12, Buckley et al, 2017 [39]*


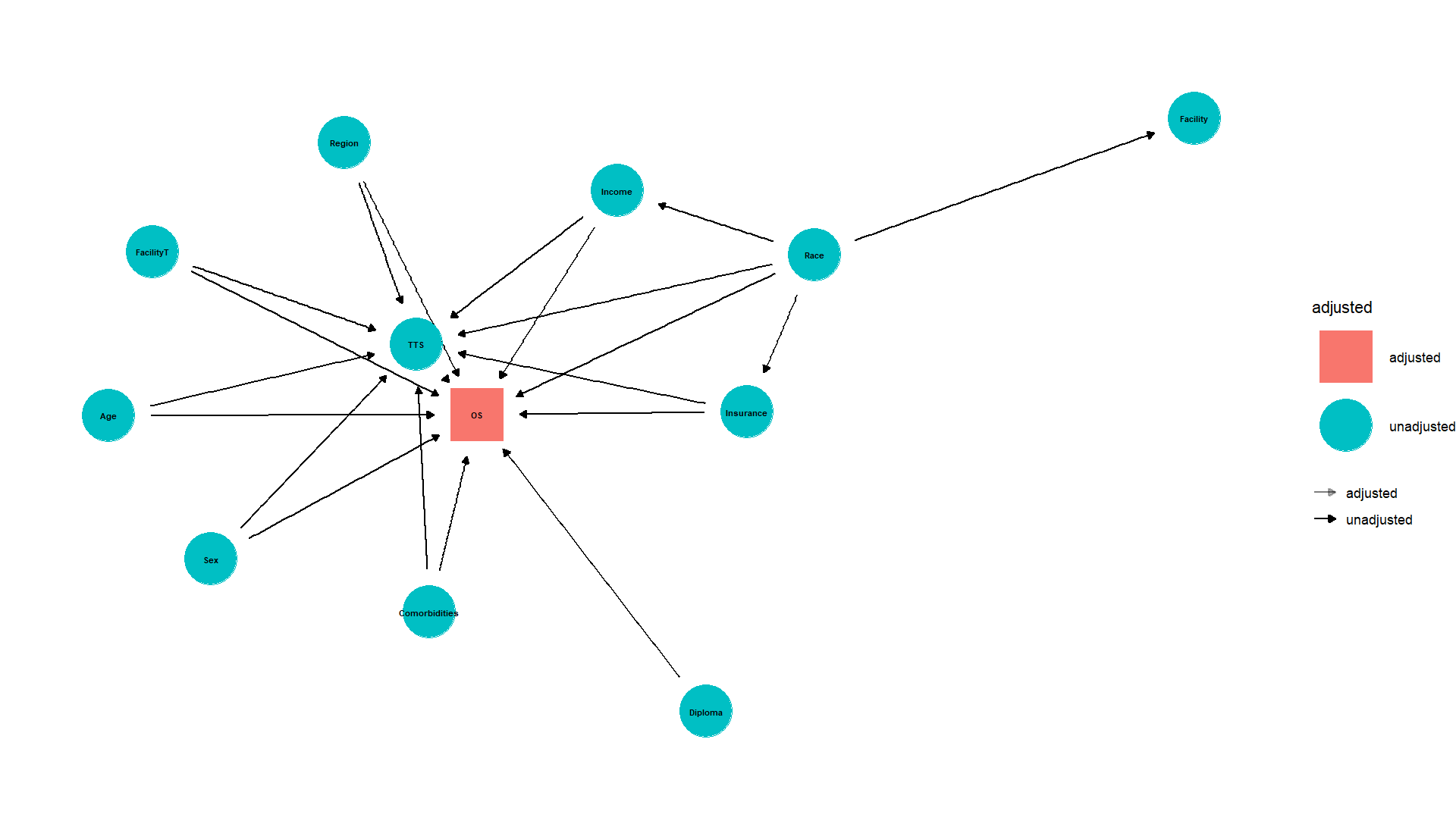


*#13: eFig13, Jabo et al 2018 [40]*


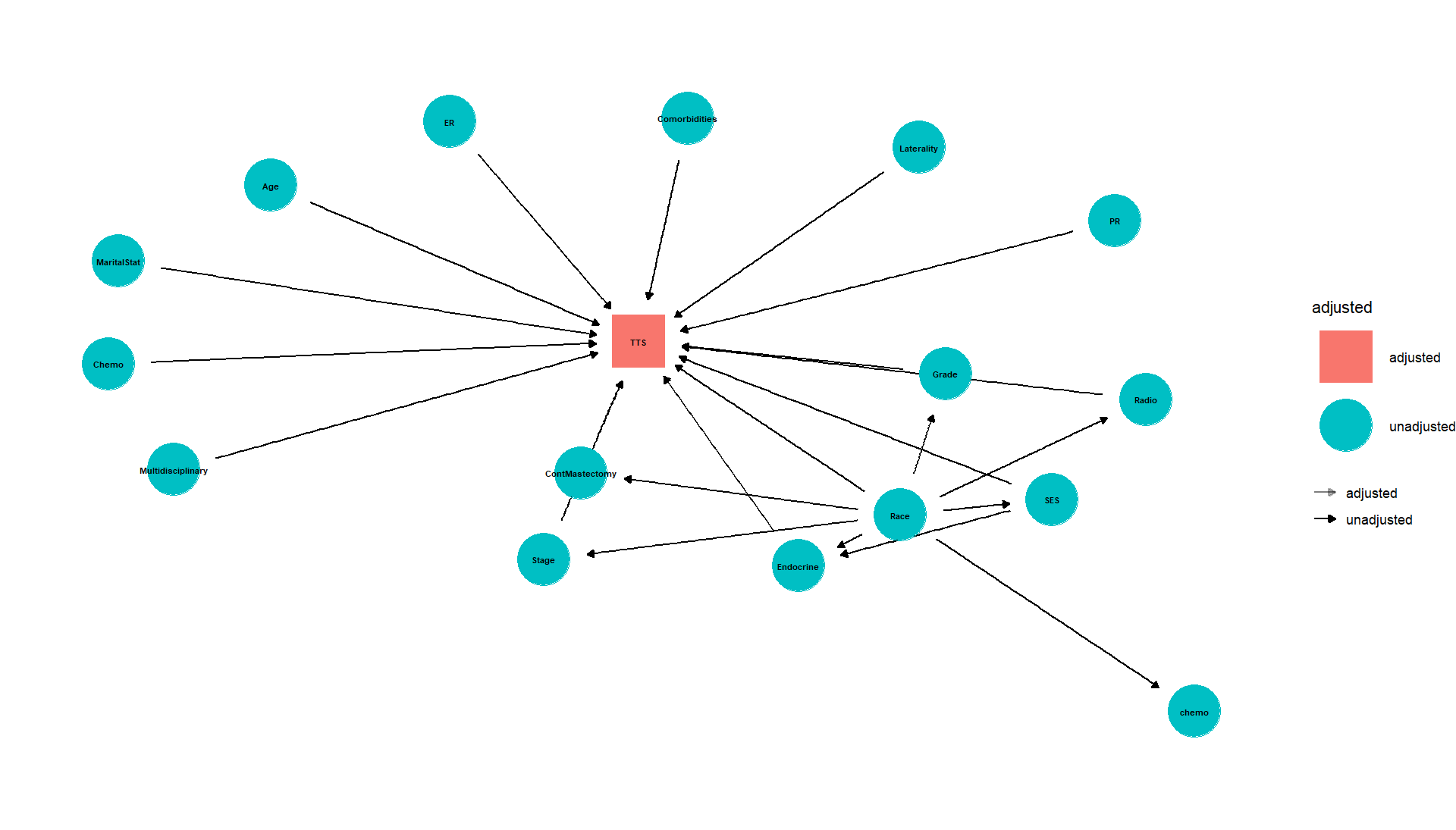


*#14: eFig14, Larson et al, 2018 [65]*


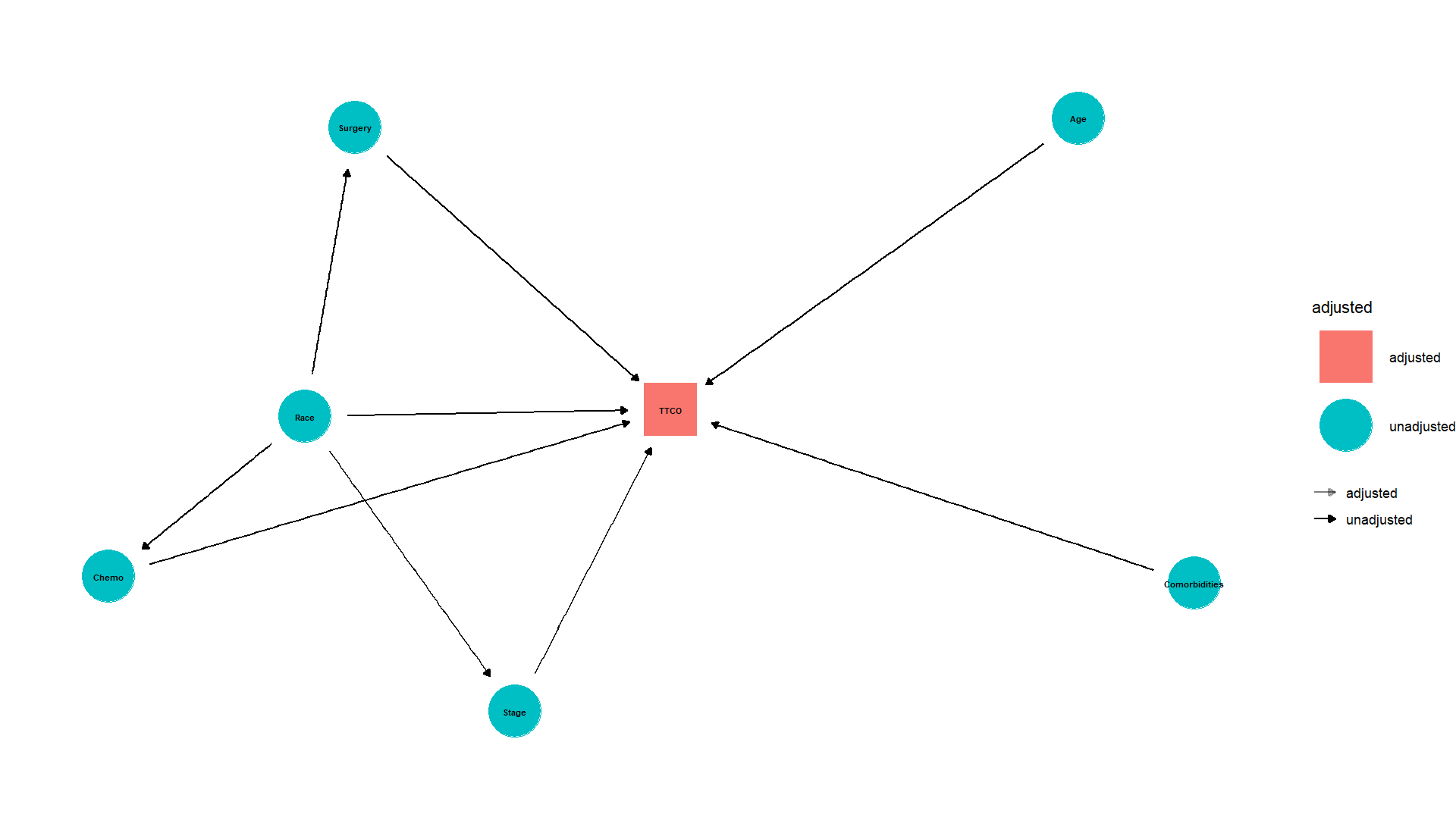


*#15:eFig15, Mariella et al, 2018 [41]*


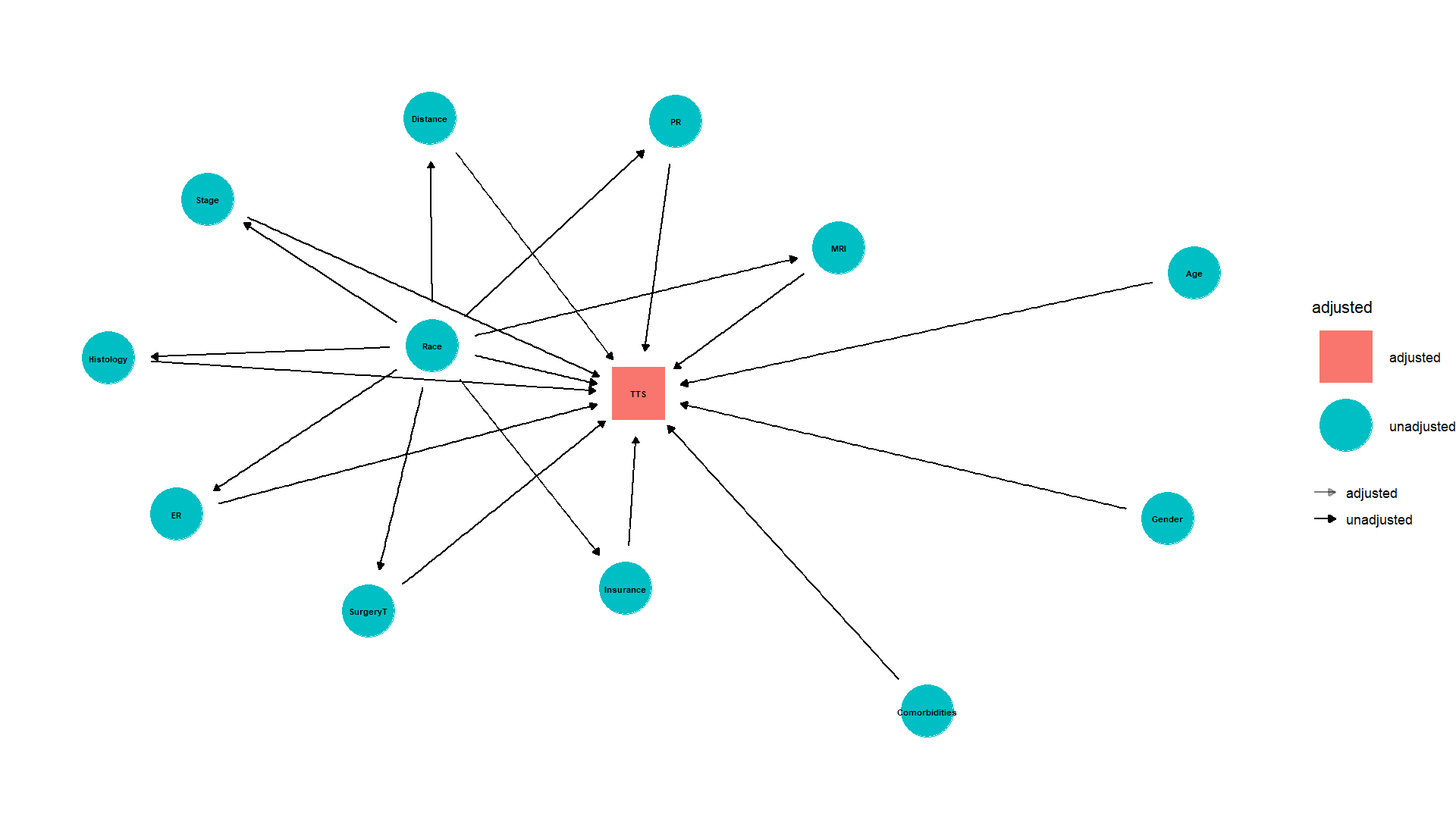


*#16:eFig16 – Jaiswal et al, 2018 [34]*


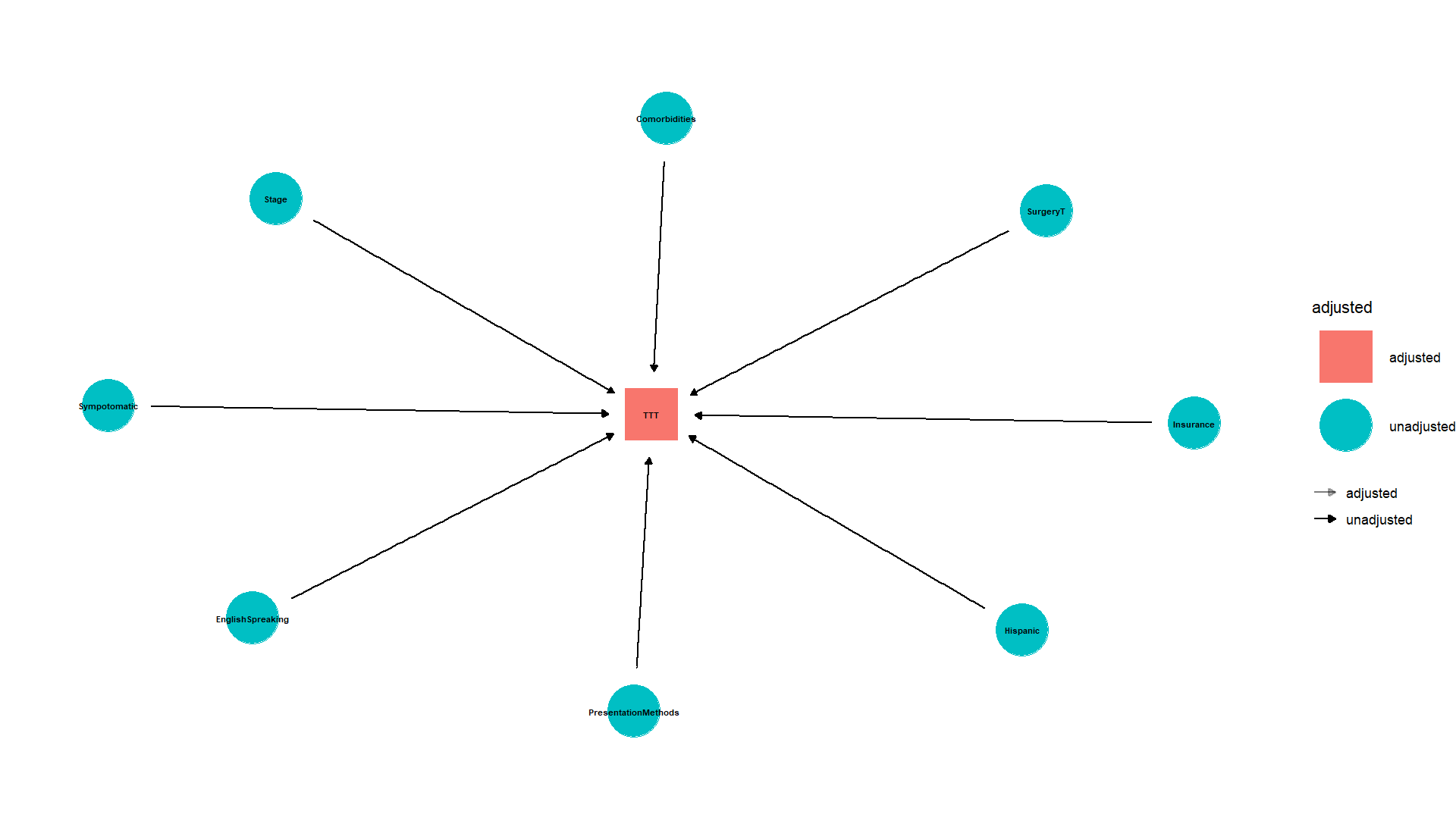


*#17:eFig17, Eaglehouse et al 2019 [42]*


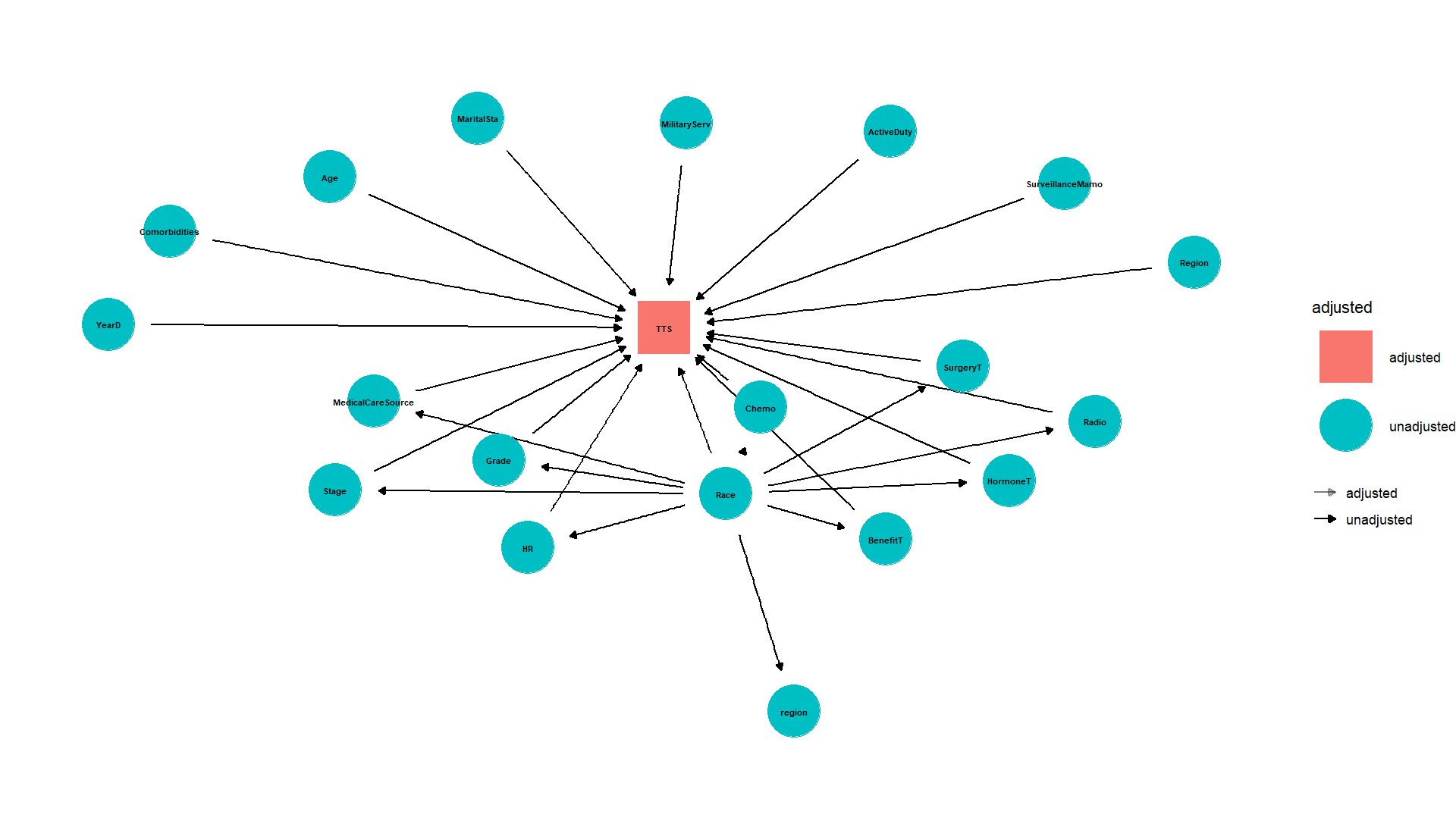


*#18: eFig18, Eaglehouse et al, 2019 [43]*


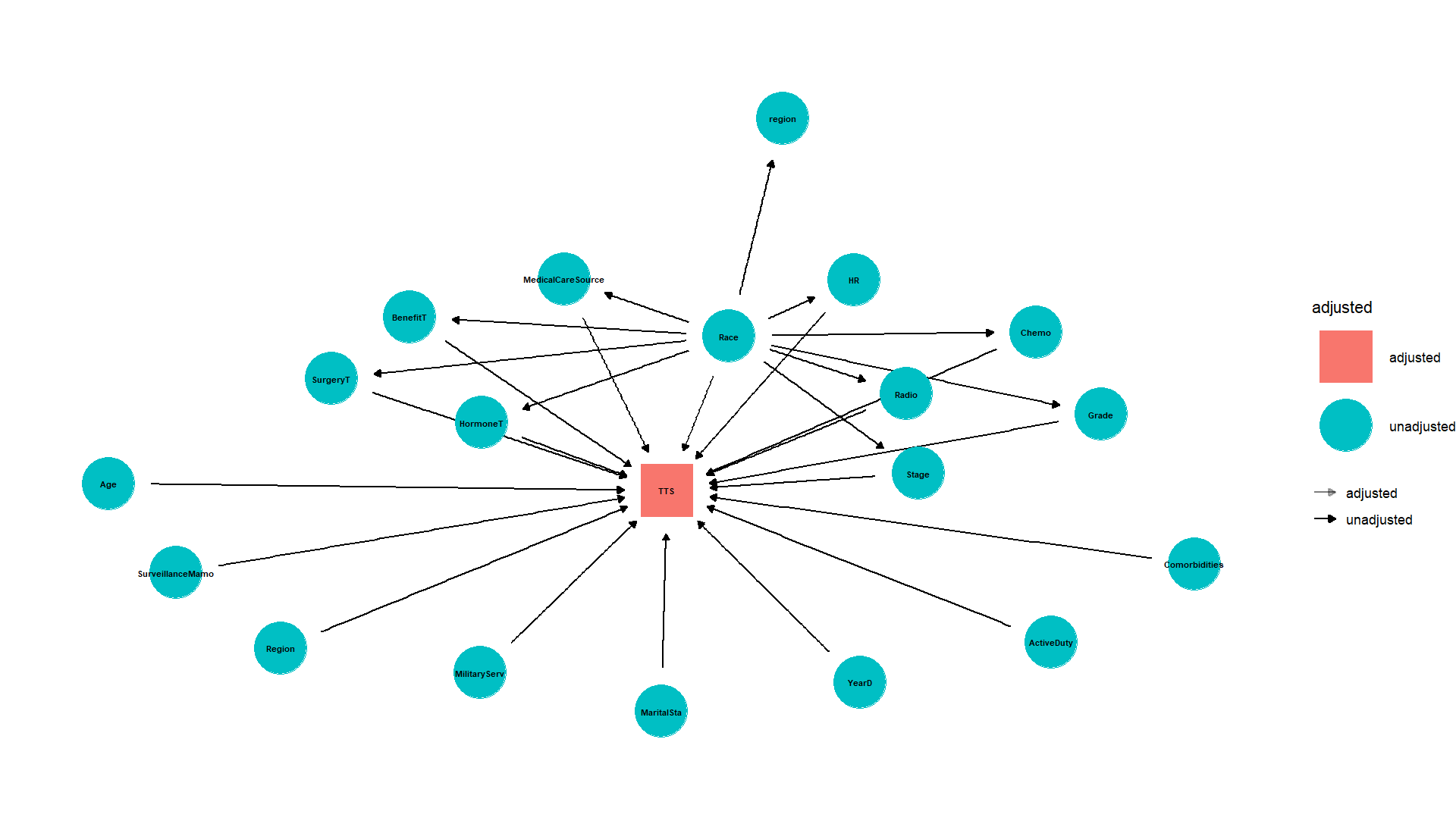


*#19: eFig19, Hoppe et al, 2019 [44]*


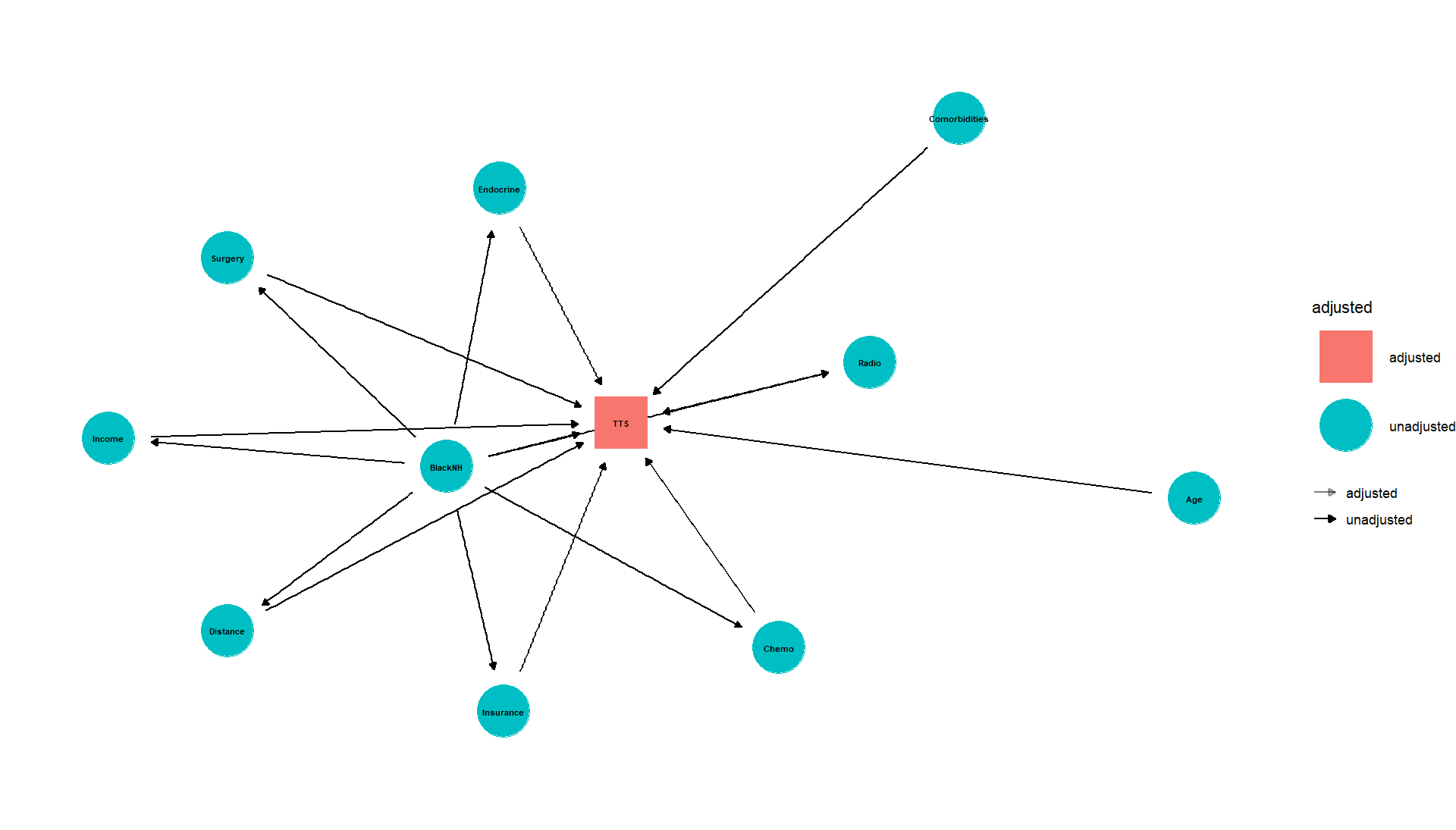


*#20: eFig20, Kupstas et al, 2019 [56]*


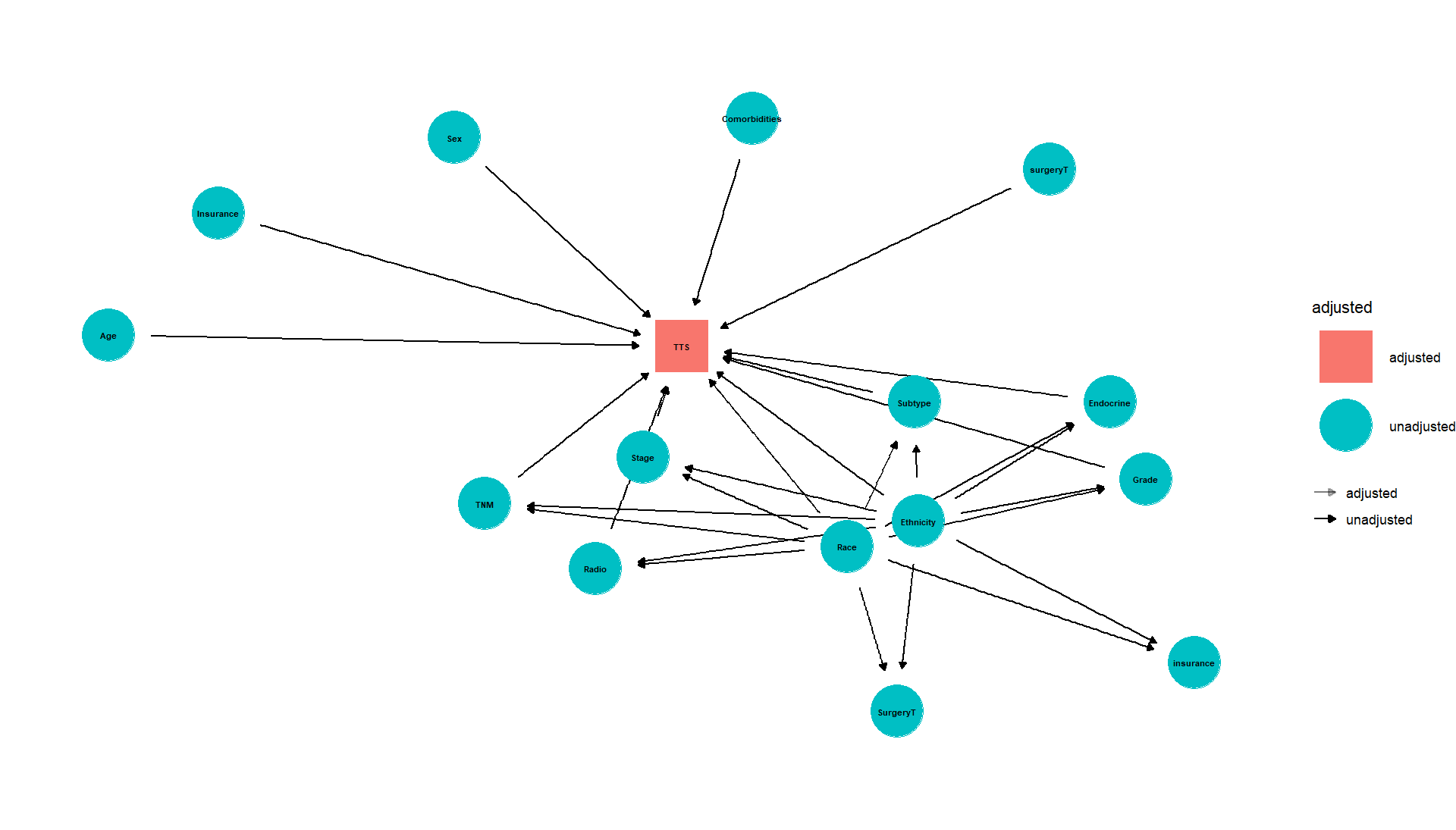


*#21:eFig21, Emerson et al, 2020 [29]*


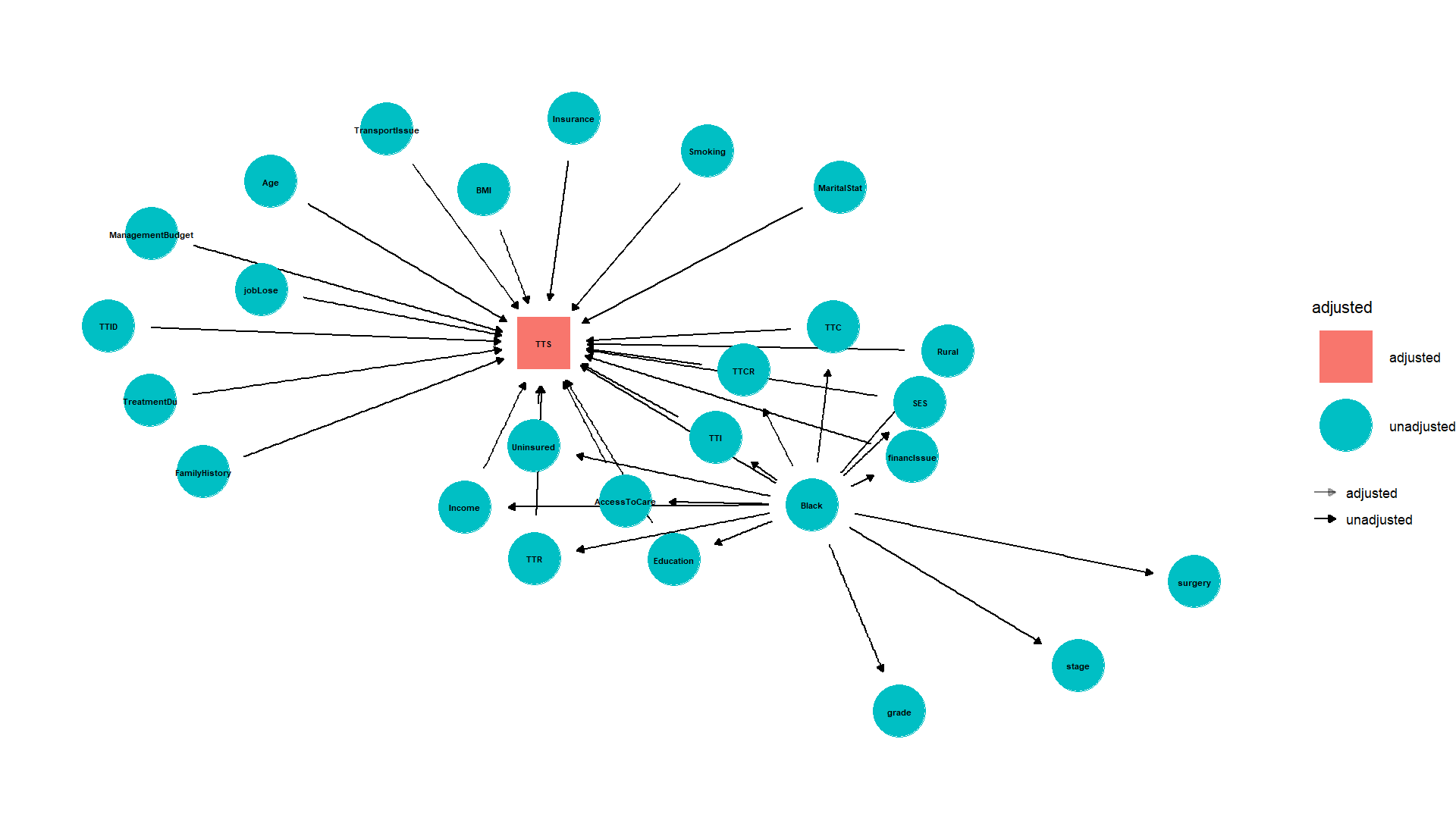


*#22: eFig22, Sutton et al 2020 [45]*


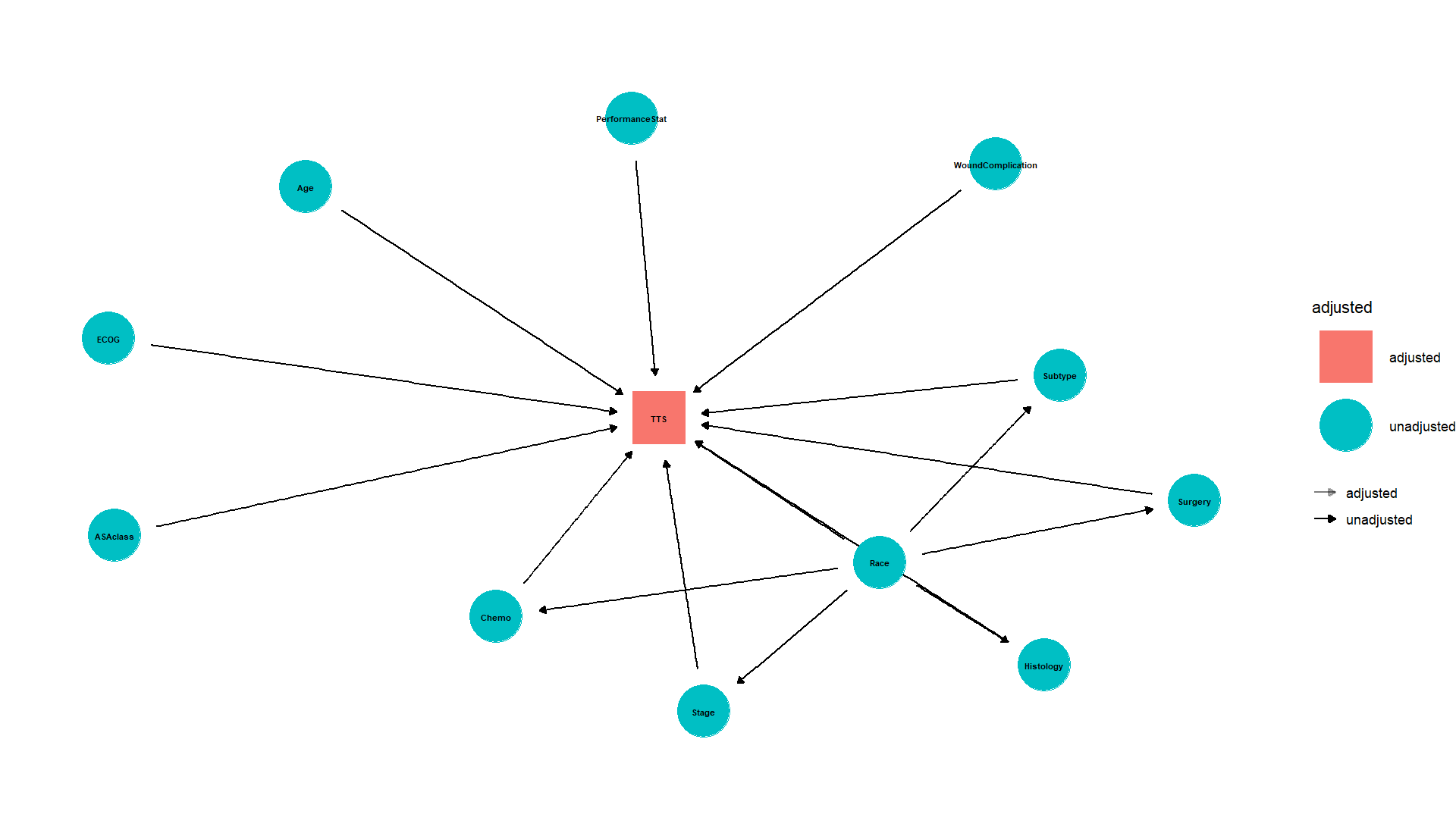


*#23: eFig23, Sutton et al 2020 [46]*


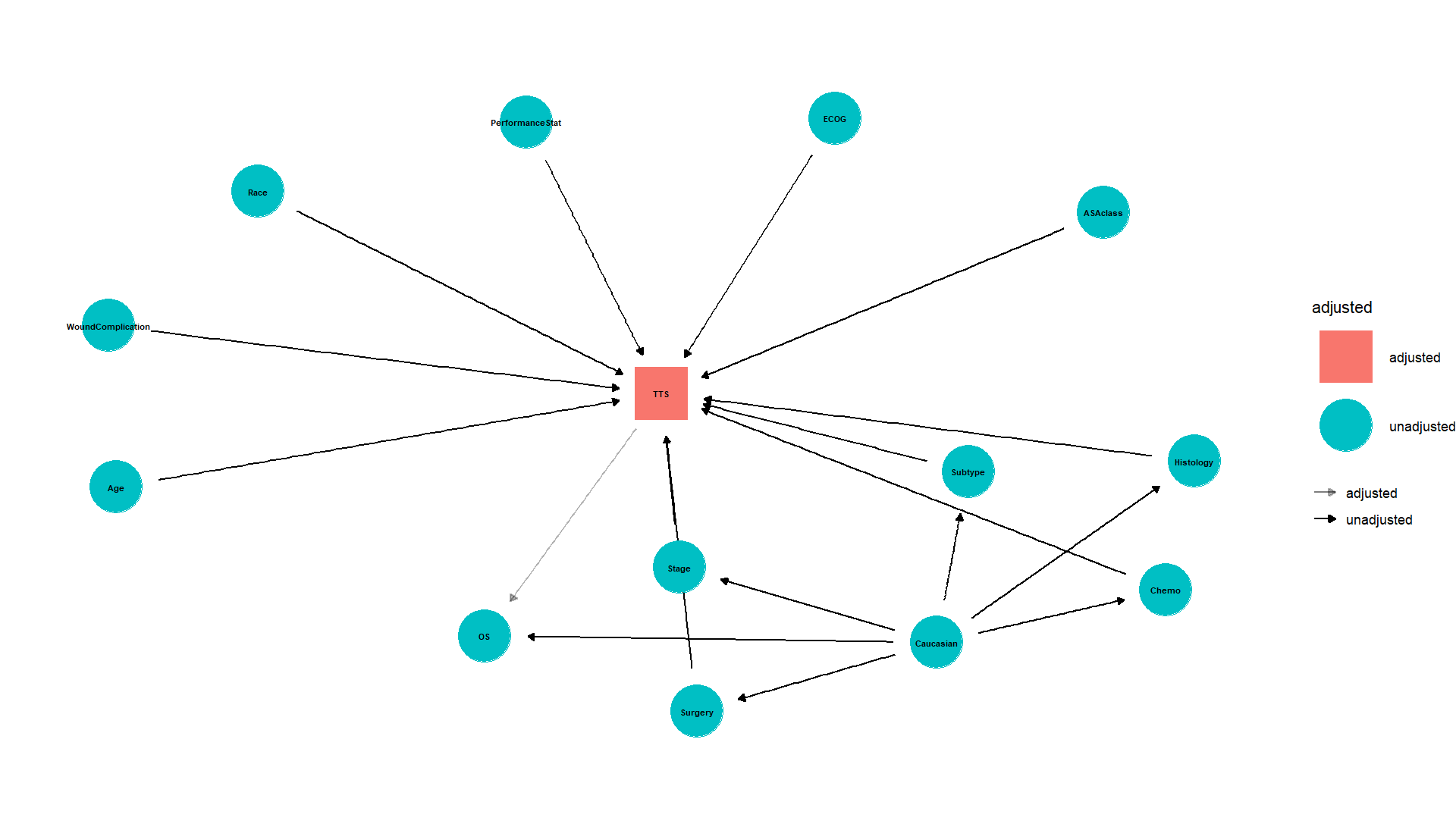


*#24:eFig24, Prakash et al, 2021 [52]*


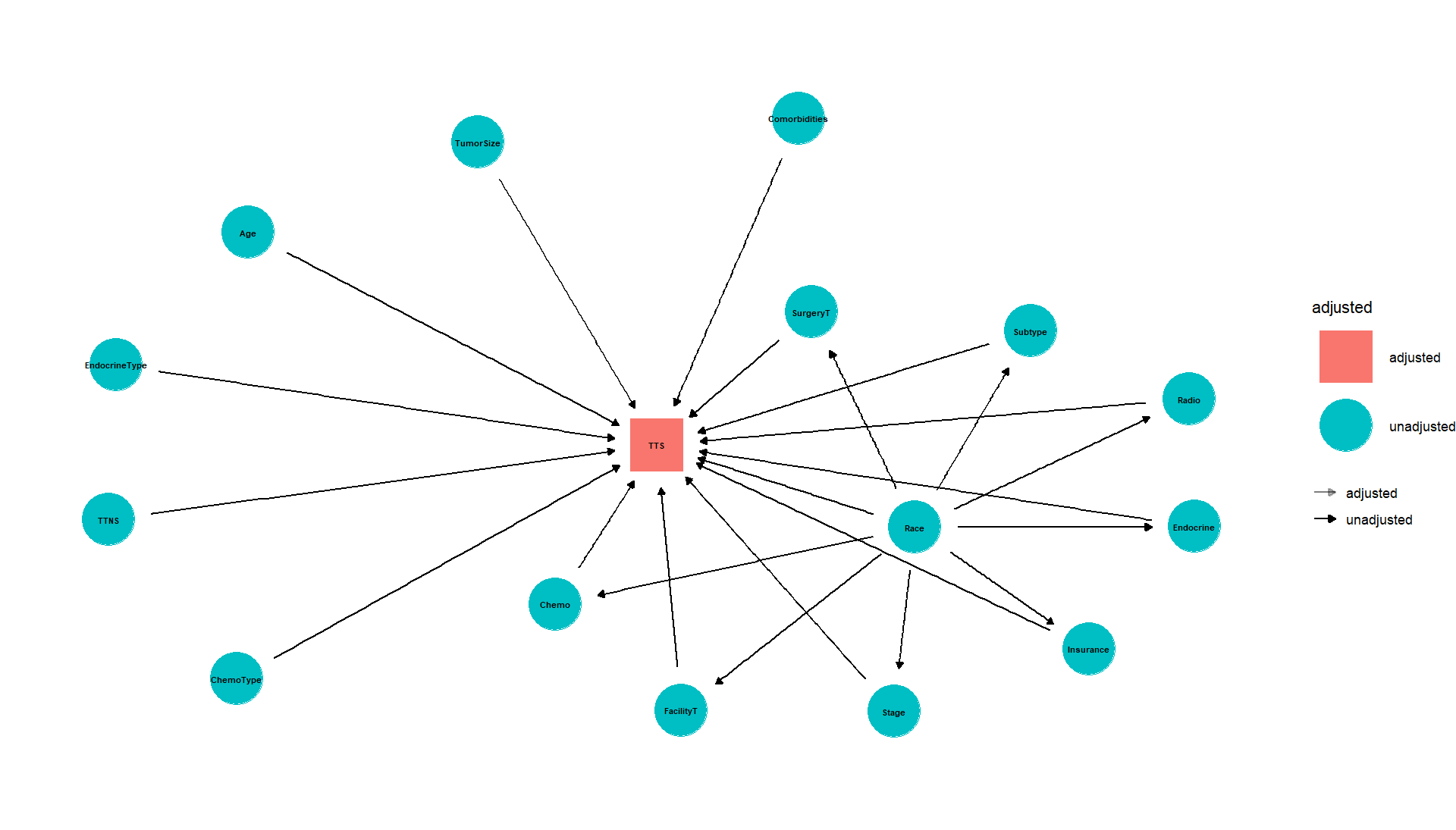


*#25:eFig25, Pratt et al, 2021 [66]*


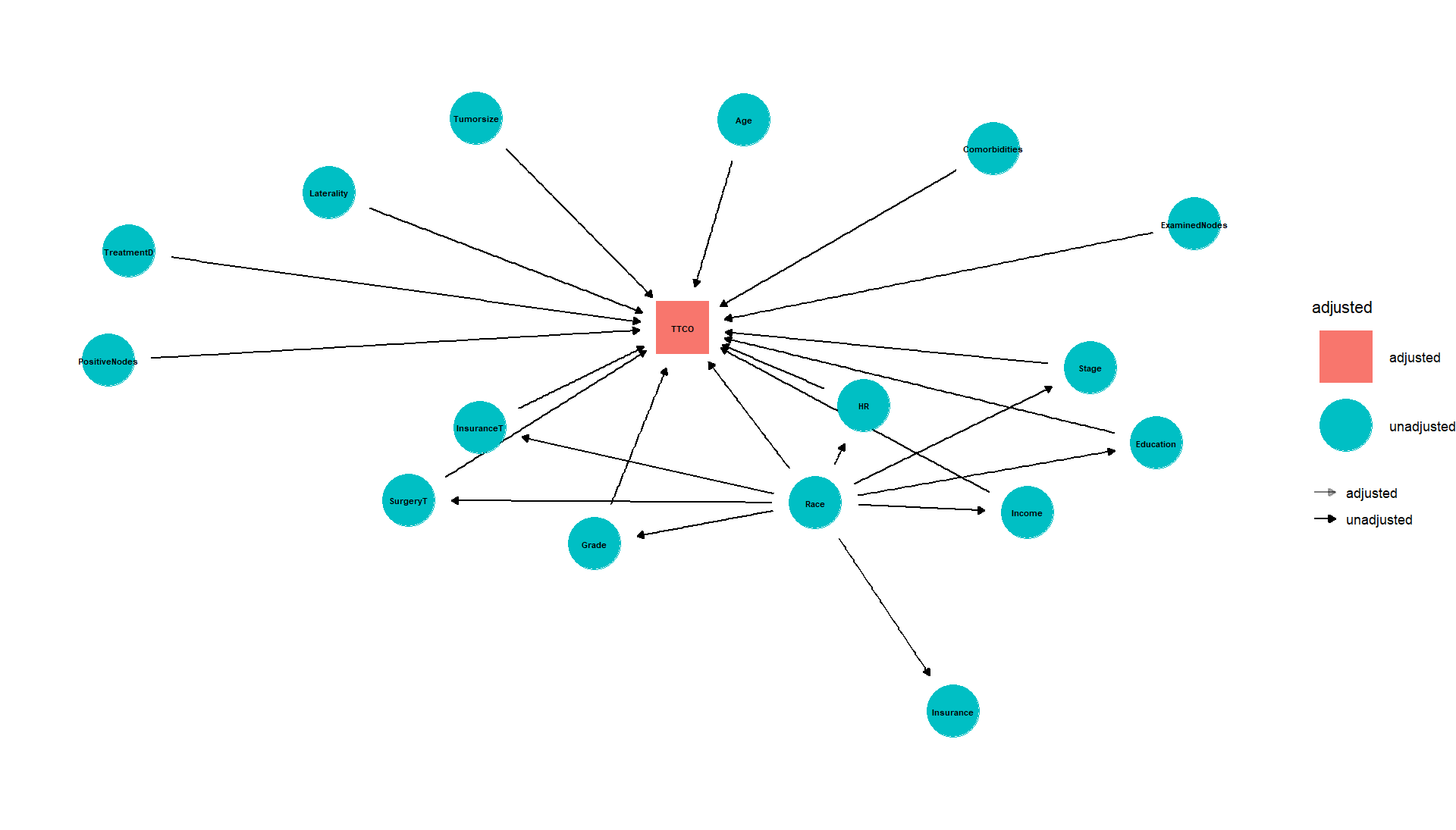


*#26:eFig26, Jackson et al, 2021 [47]*


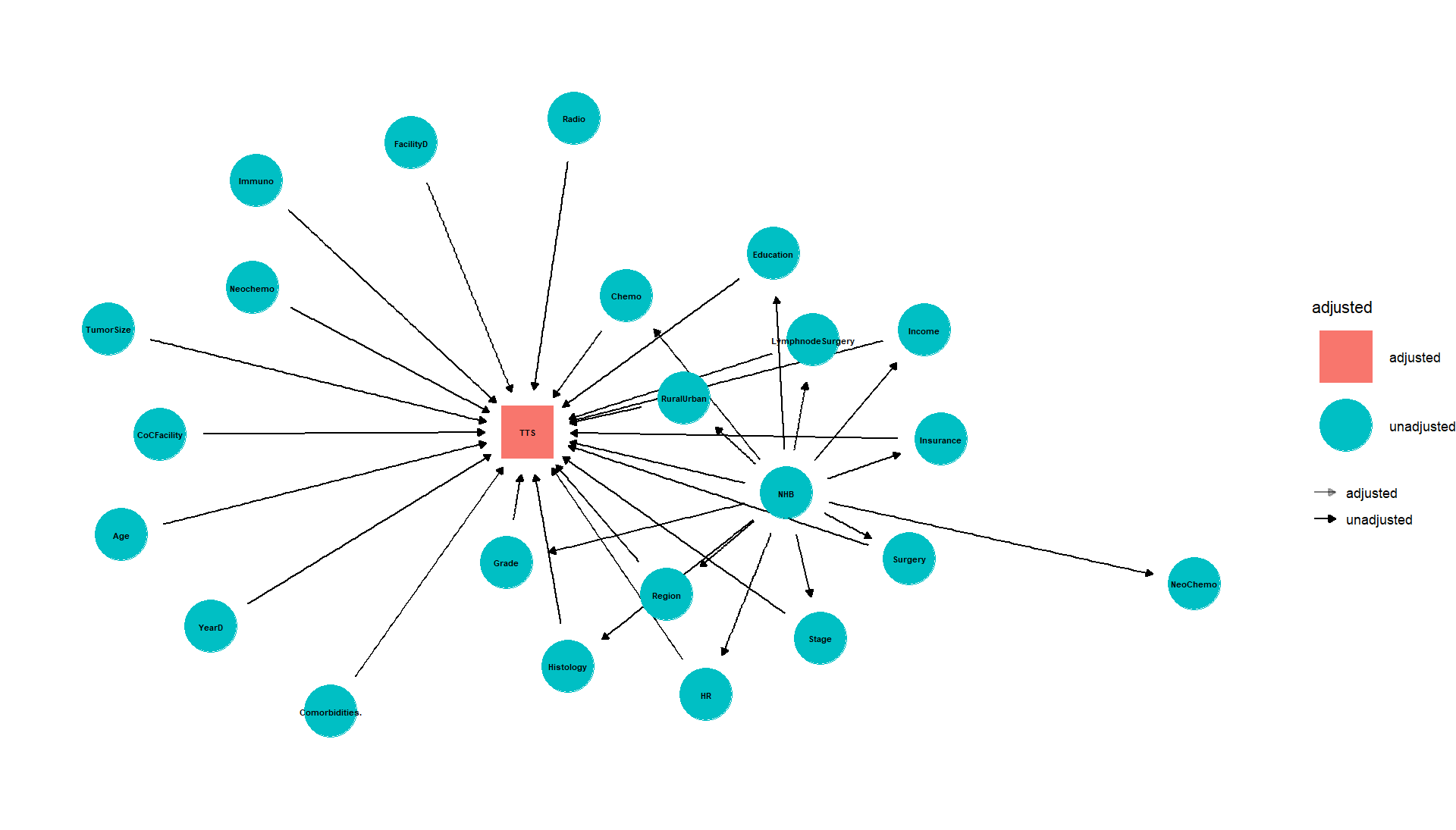


*#27:eFig27, Dankwa Mullan et al, 2021 [48]*


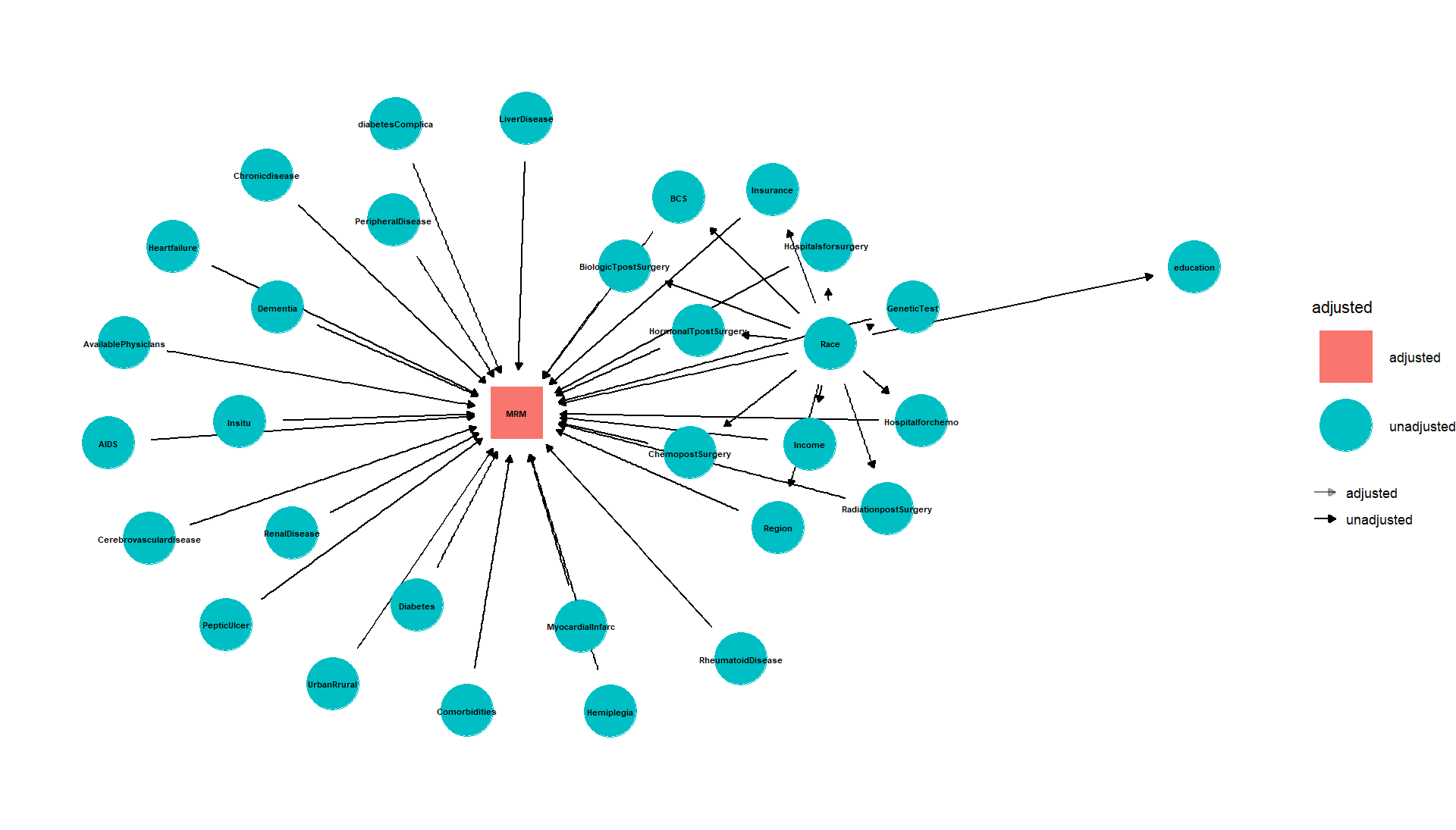


*#28:eFig28, Blazek et al, 2021 [62]*


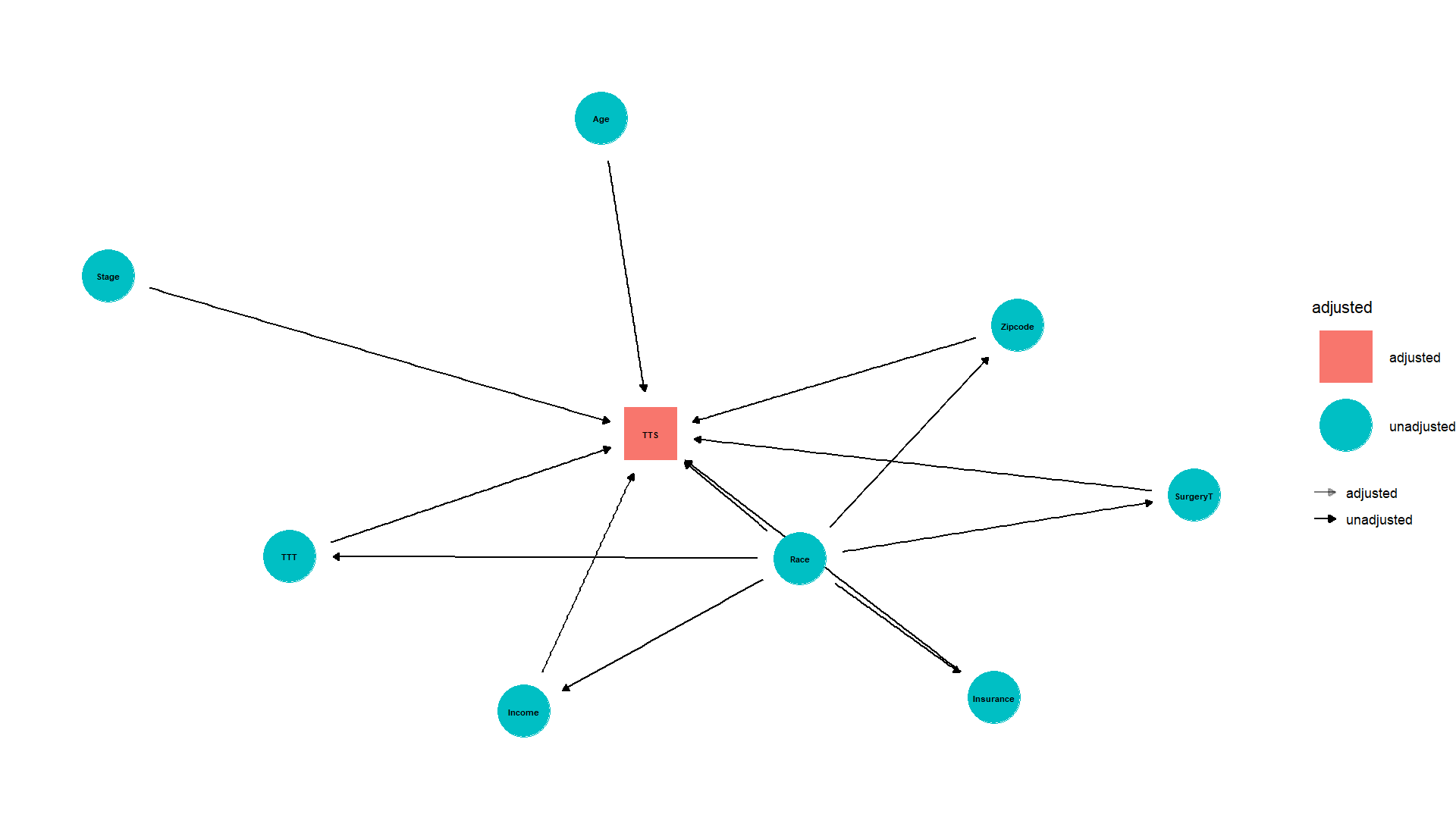


*#29:eFig29, Chagpar et al, 2022 [49]*


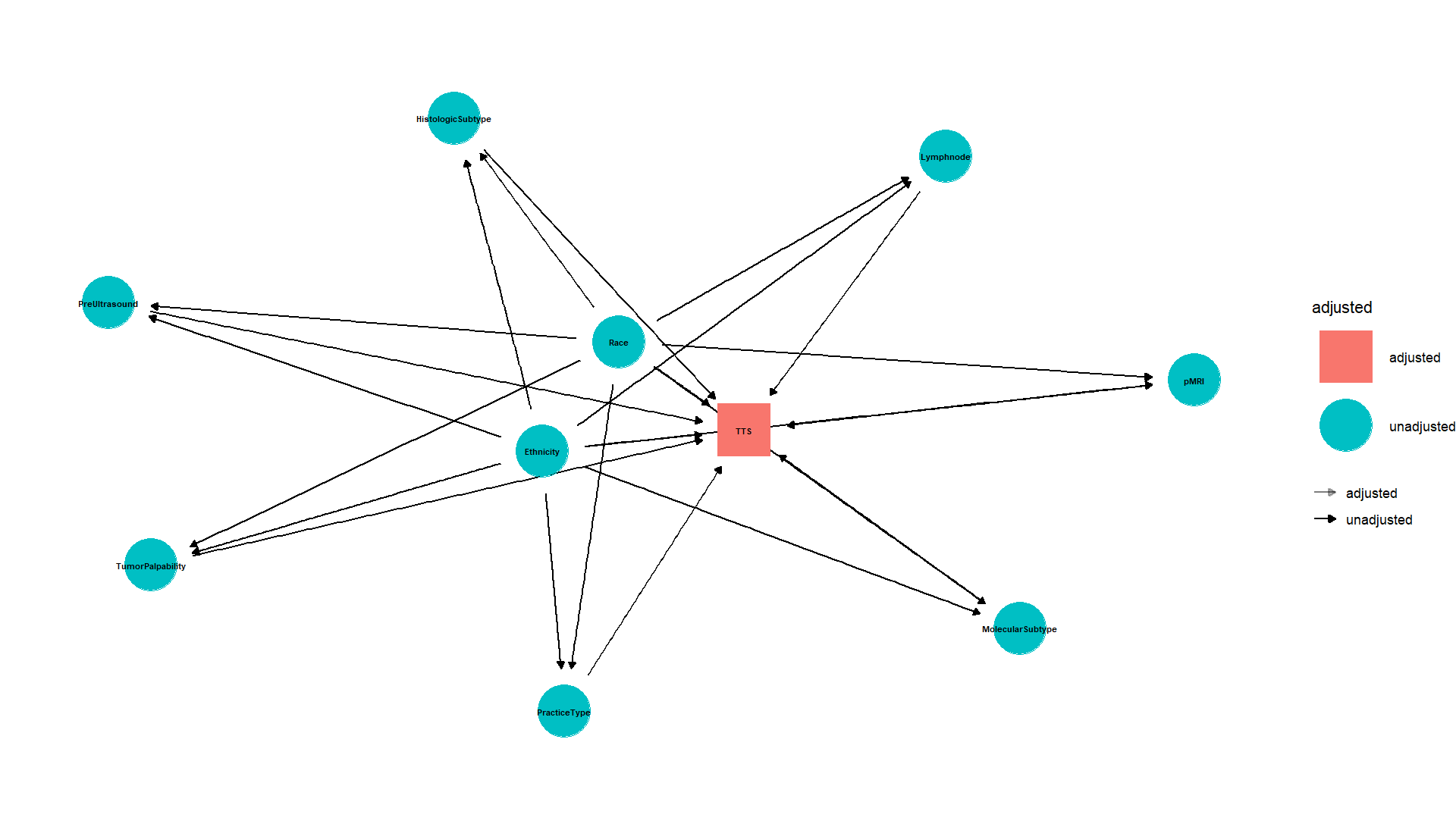


*#30:eFig30, Navarro et al 2022 [50]*


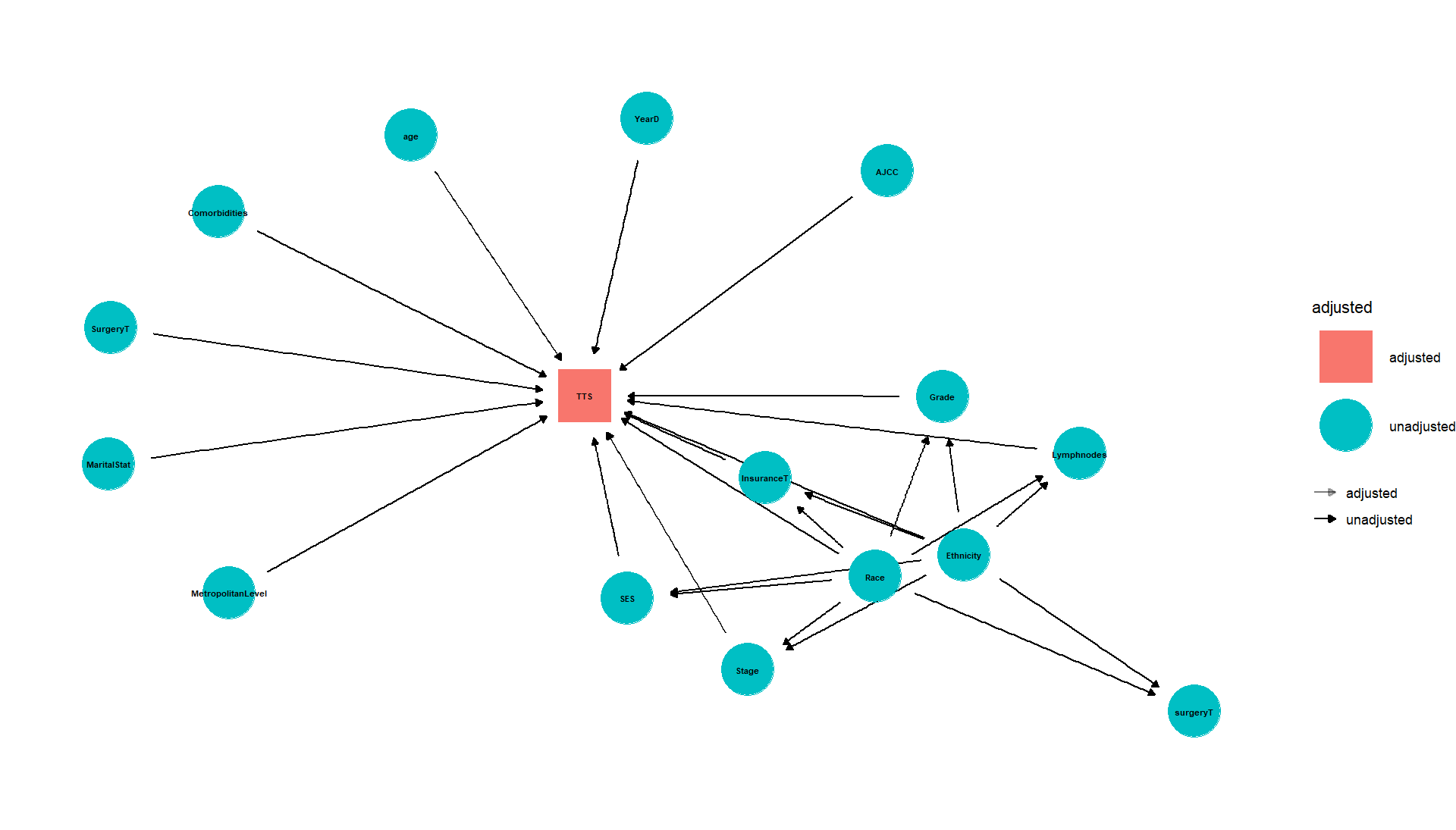


*#31: eFig31, Schermerhorn et al, 2022 [30]*


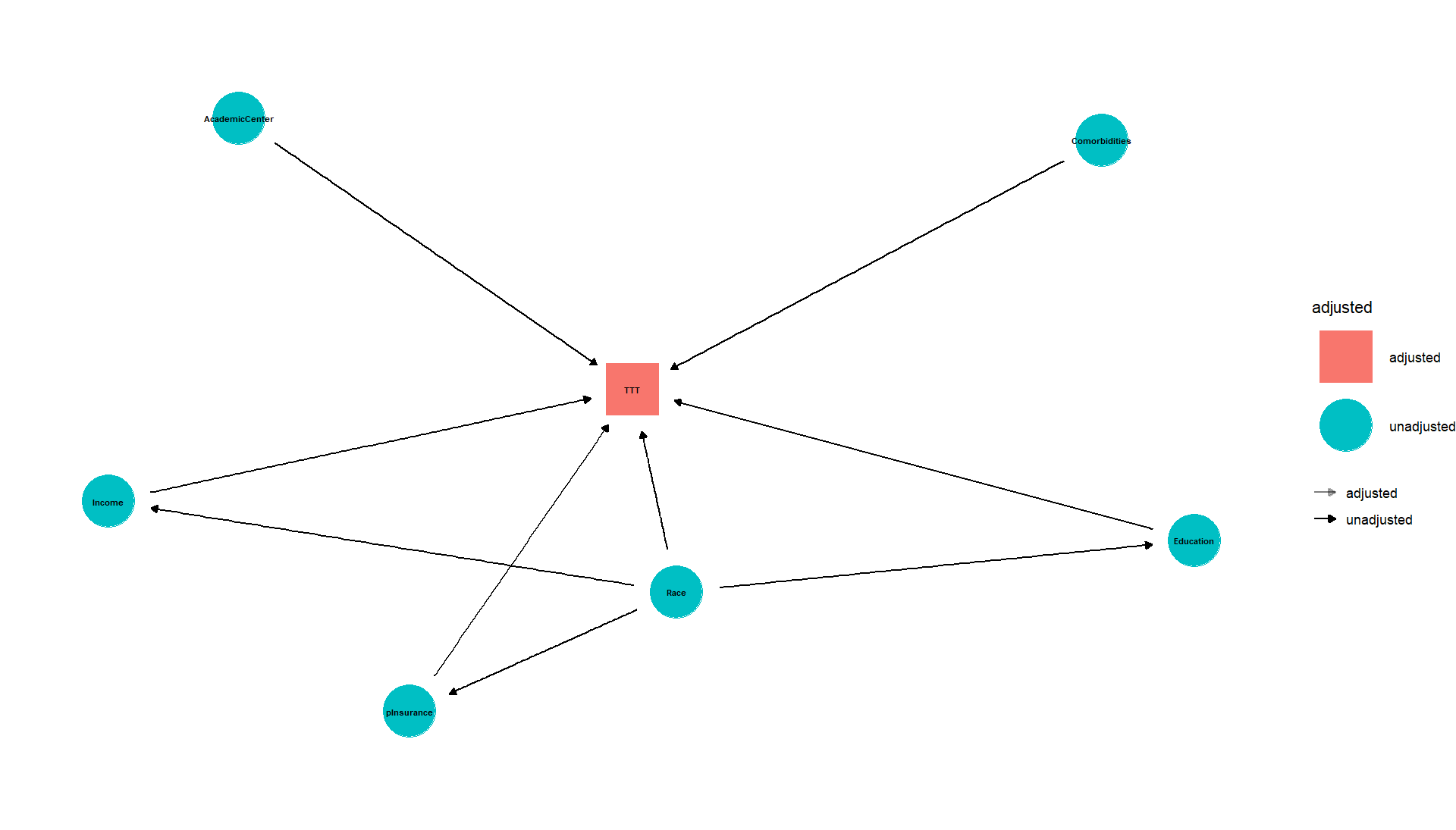


*#32: eFig32, Zaveri et al 2022 [31]*


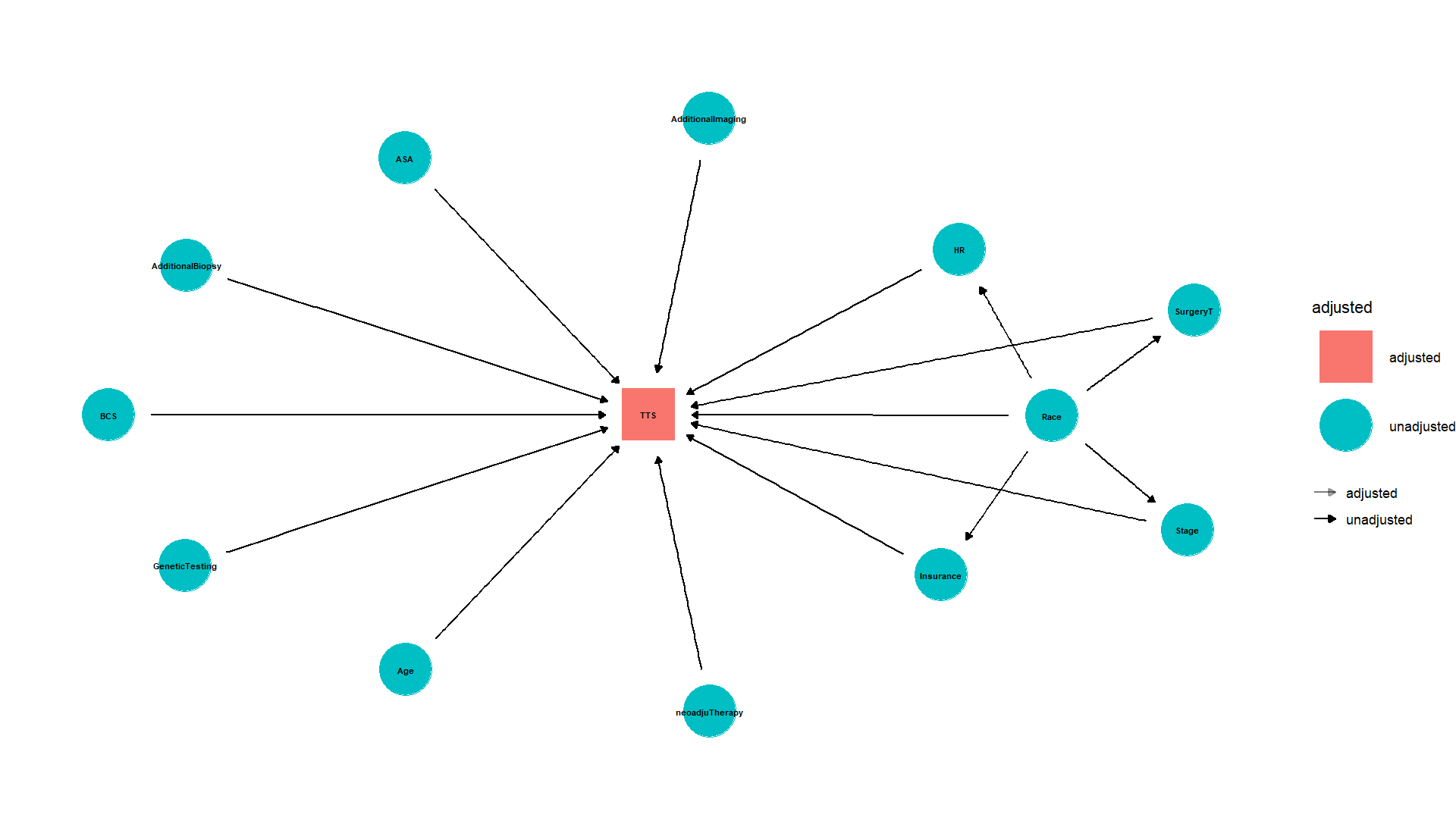


*#33:eFig33, Sukniam et al, 2022 [32]*


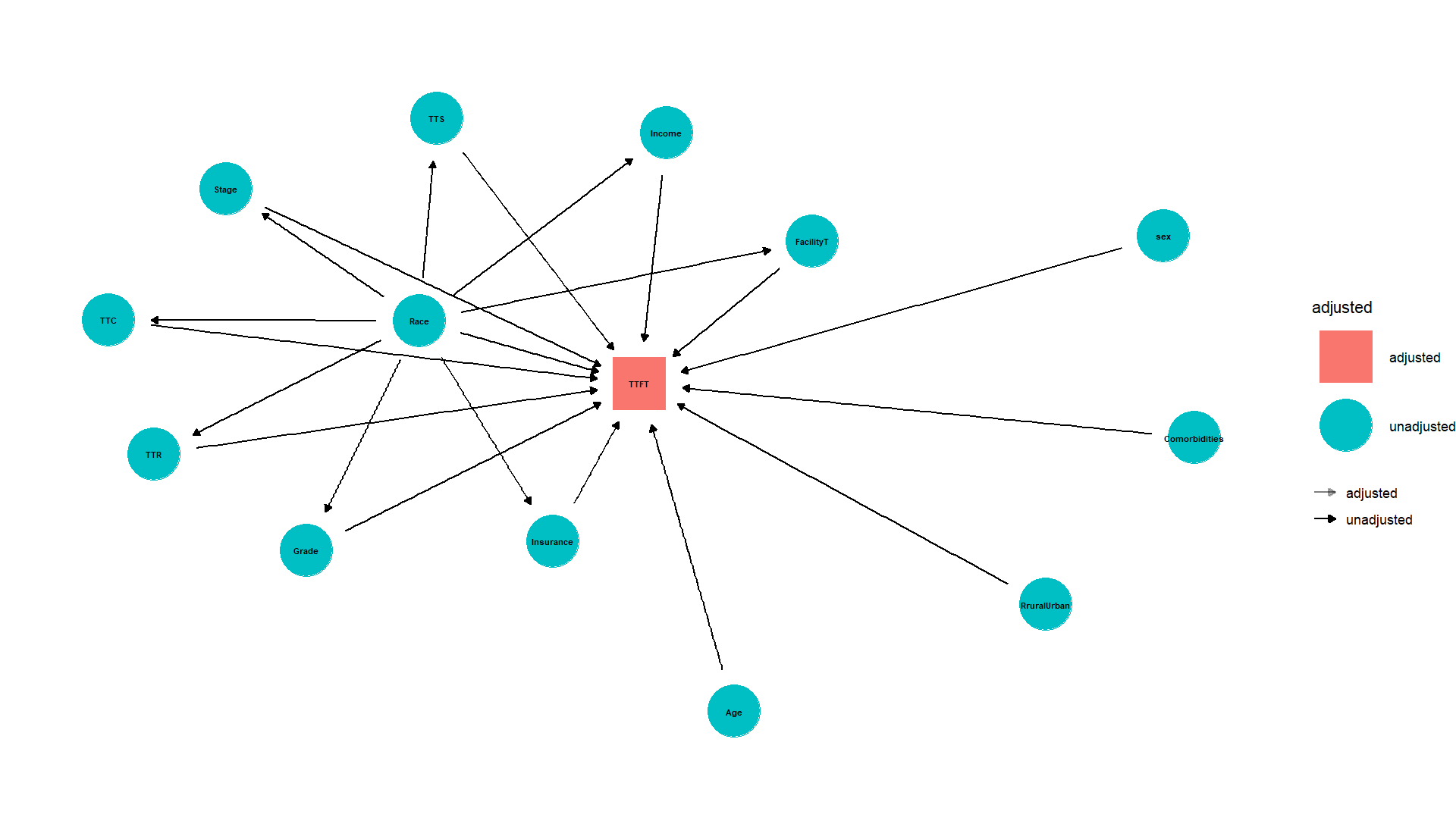


*#34:eFig34, Taparra et al, 2022 [51]*


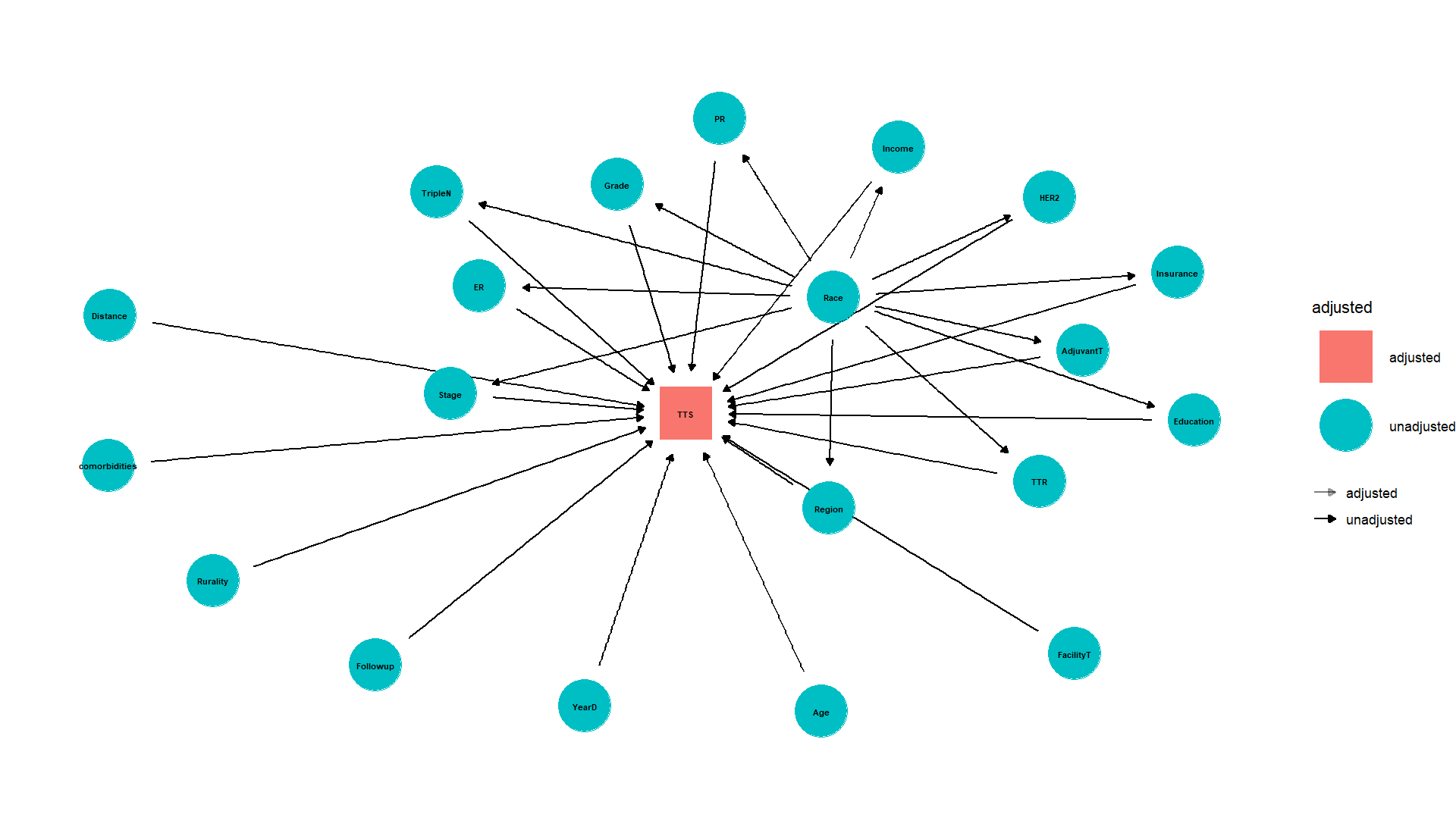


*#35:eFig35, Tjoe et al, 2022 [53]*


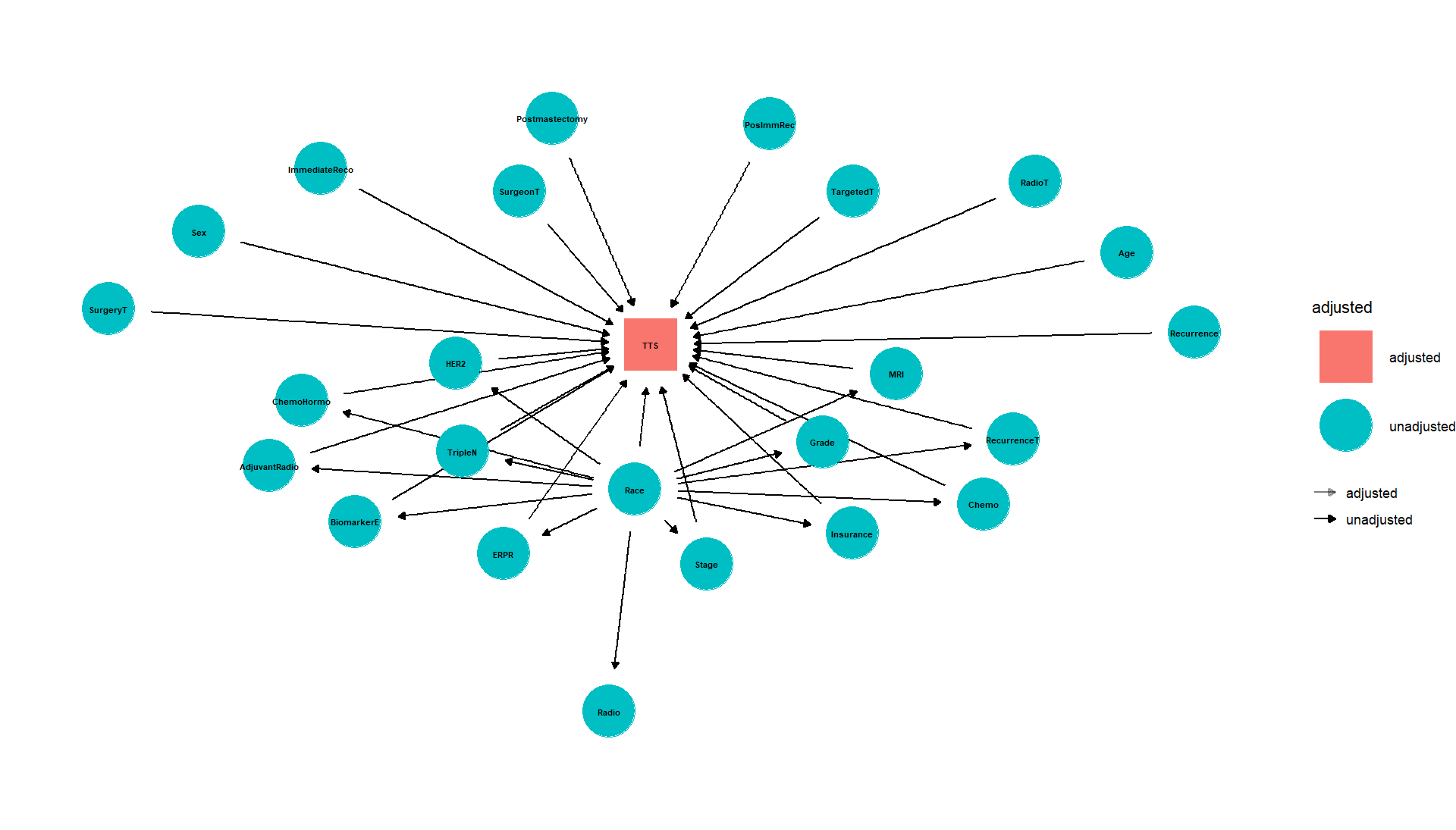


*#36:eFig36, Verdone et al, 2022 [54]*


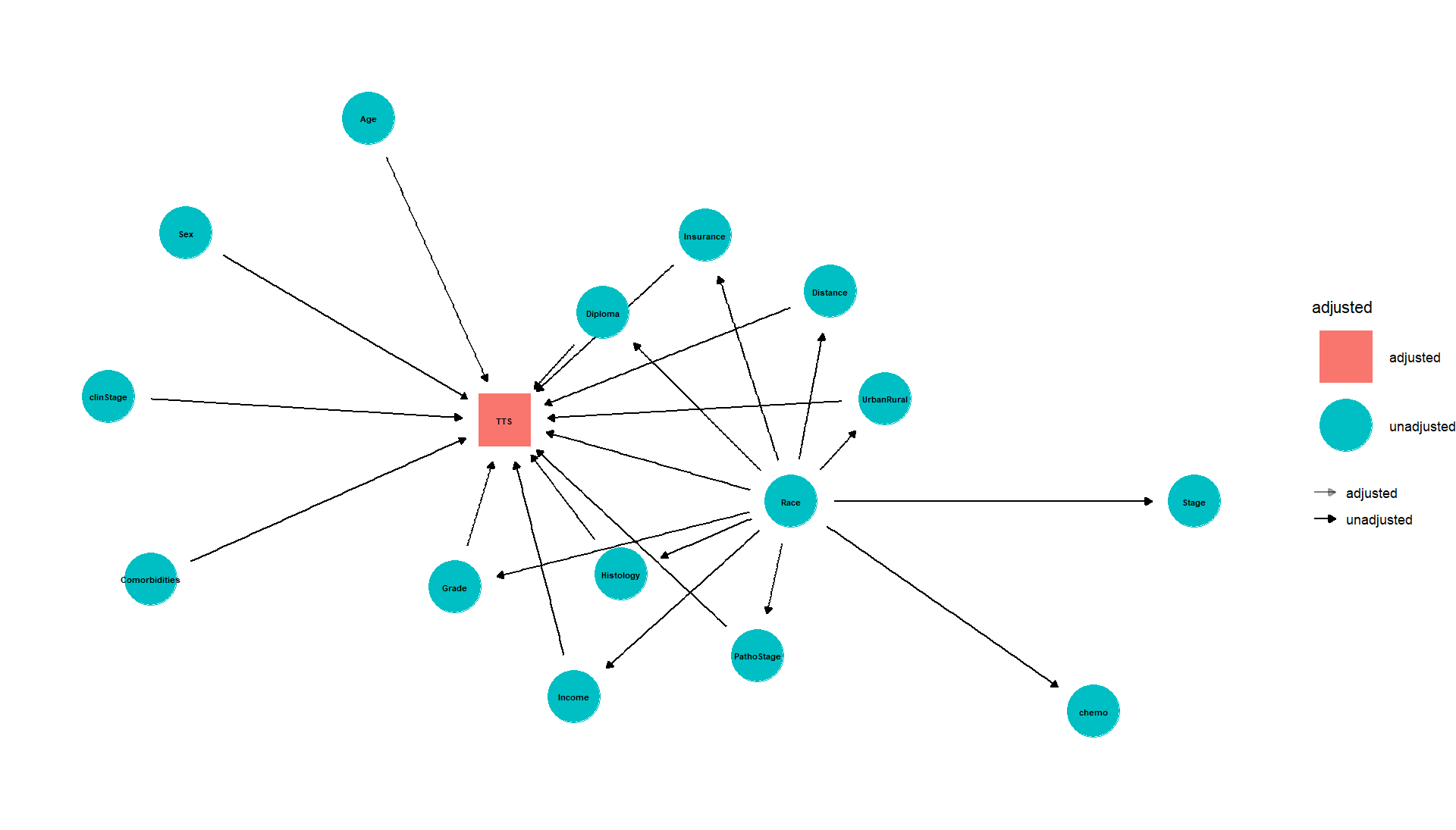


*#37:eFig37, Chen et al, 2023 [57]*


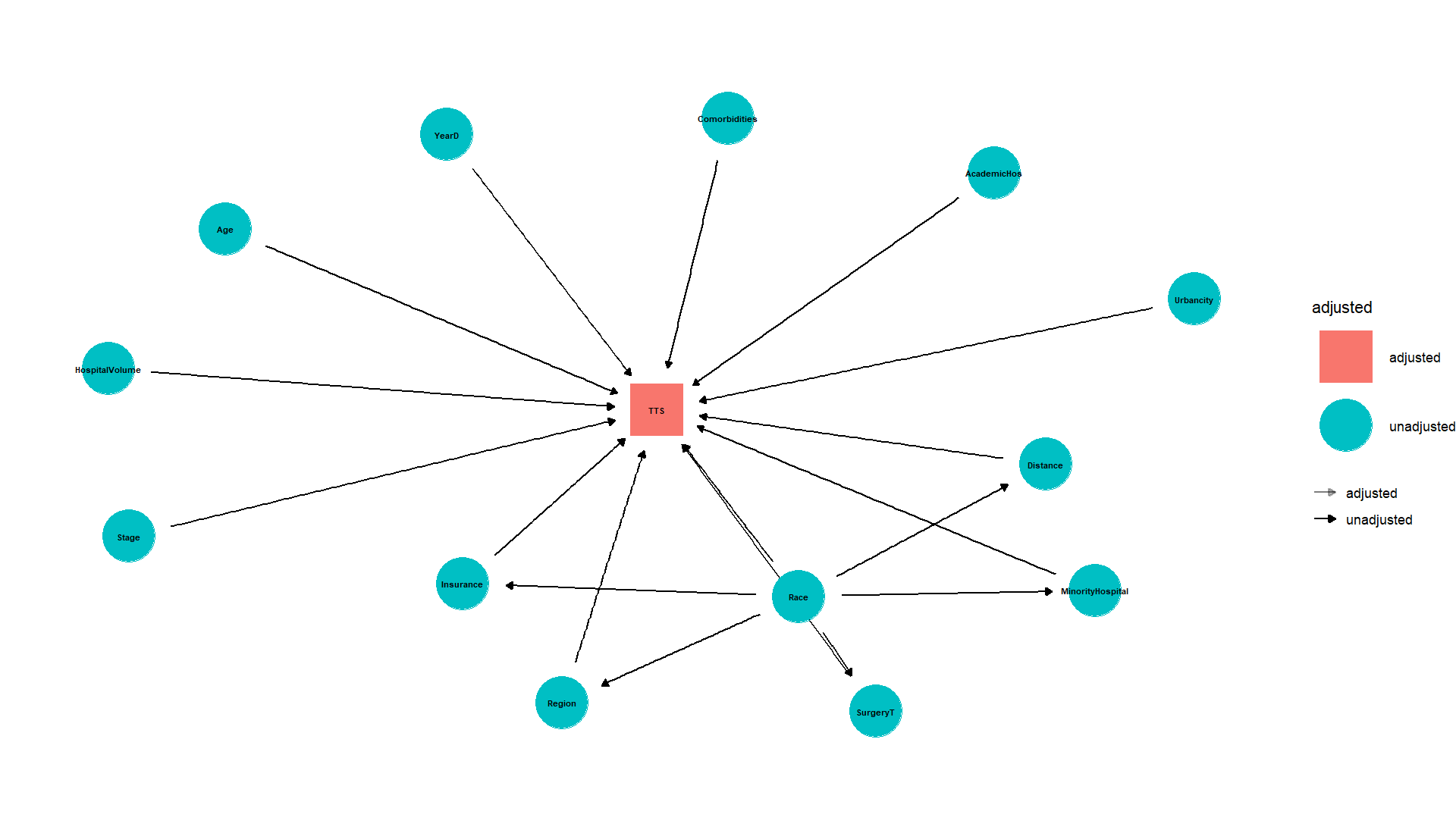


*#38:eFig38, Patel et al, 2023 [58]*


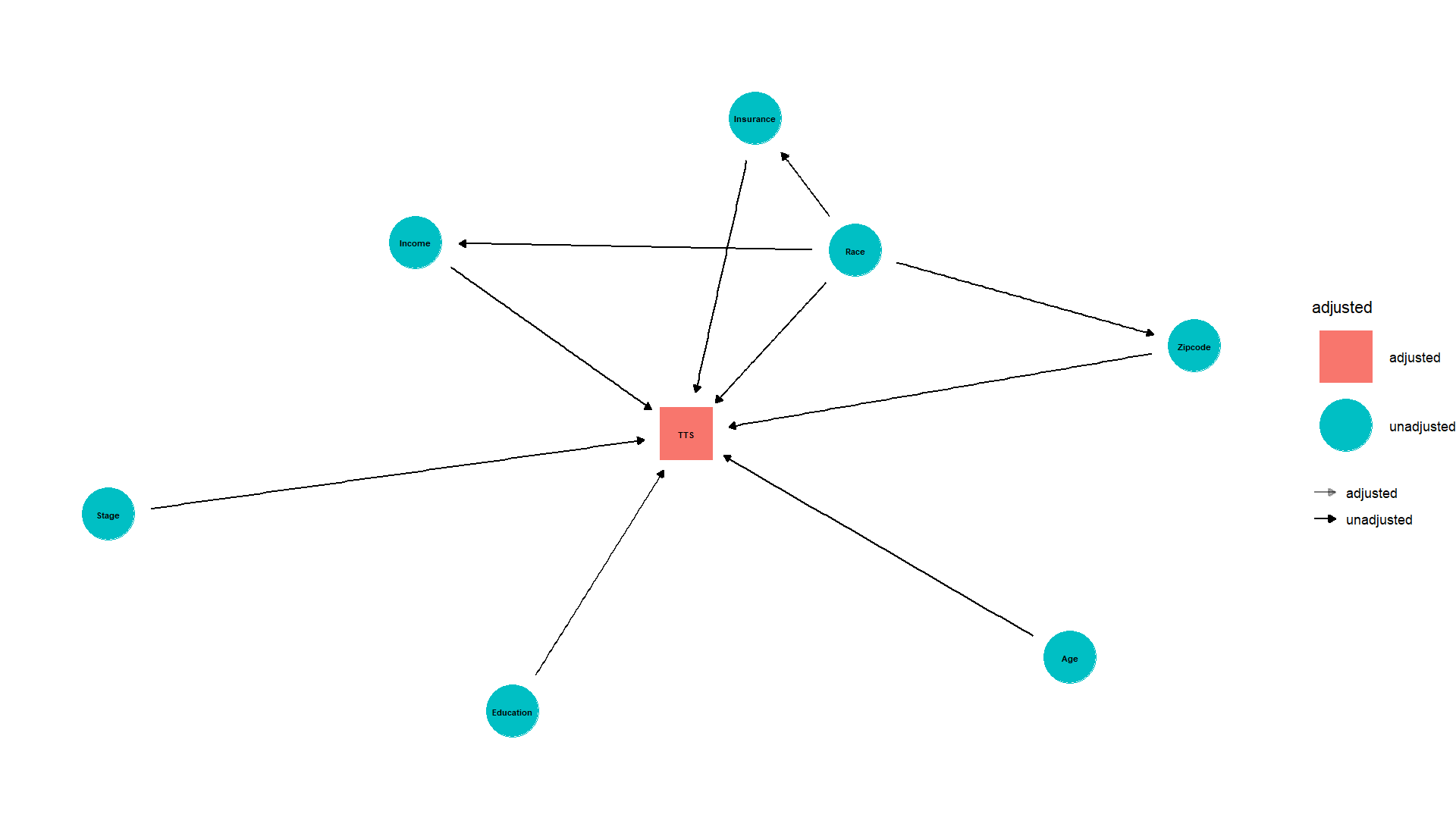


*#39: eFig39, Chavez-MacGregor et al, 2023 [64]*


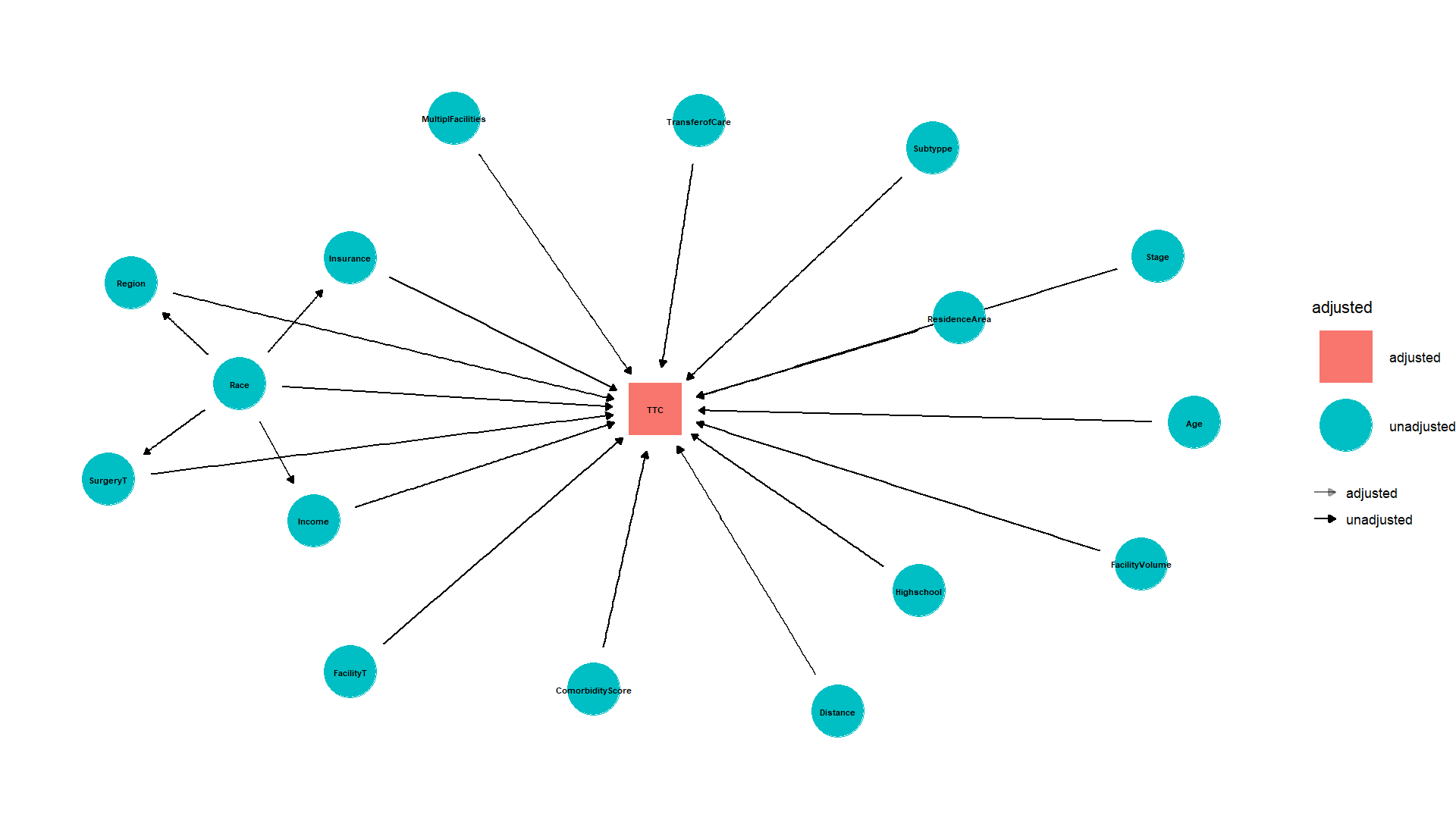


*#40: eFig40, Beaulieu-Jones et al 2024 [33]*


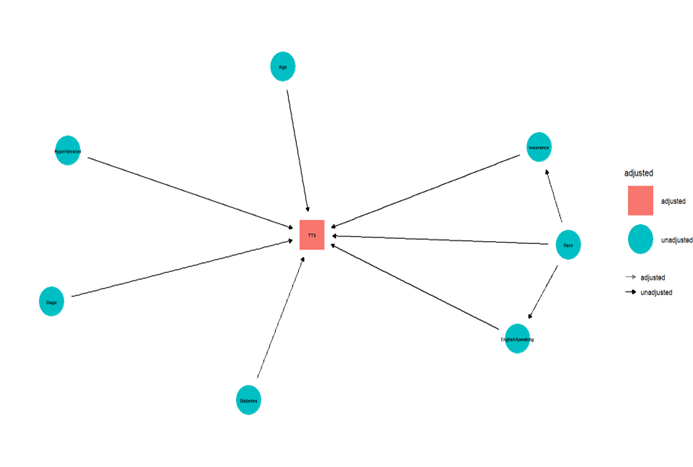


Figure S41: Samples sizes and study time periods of different databases in included studies


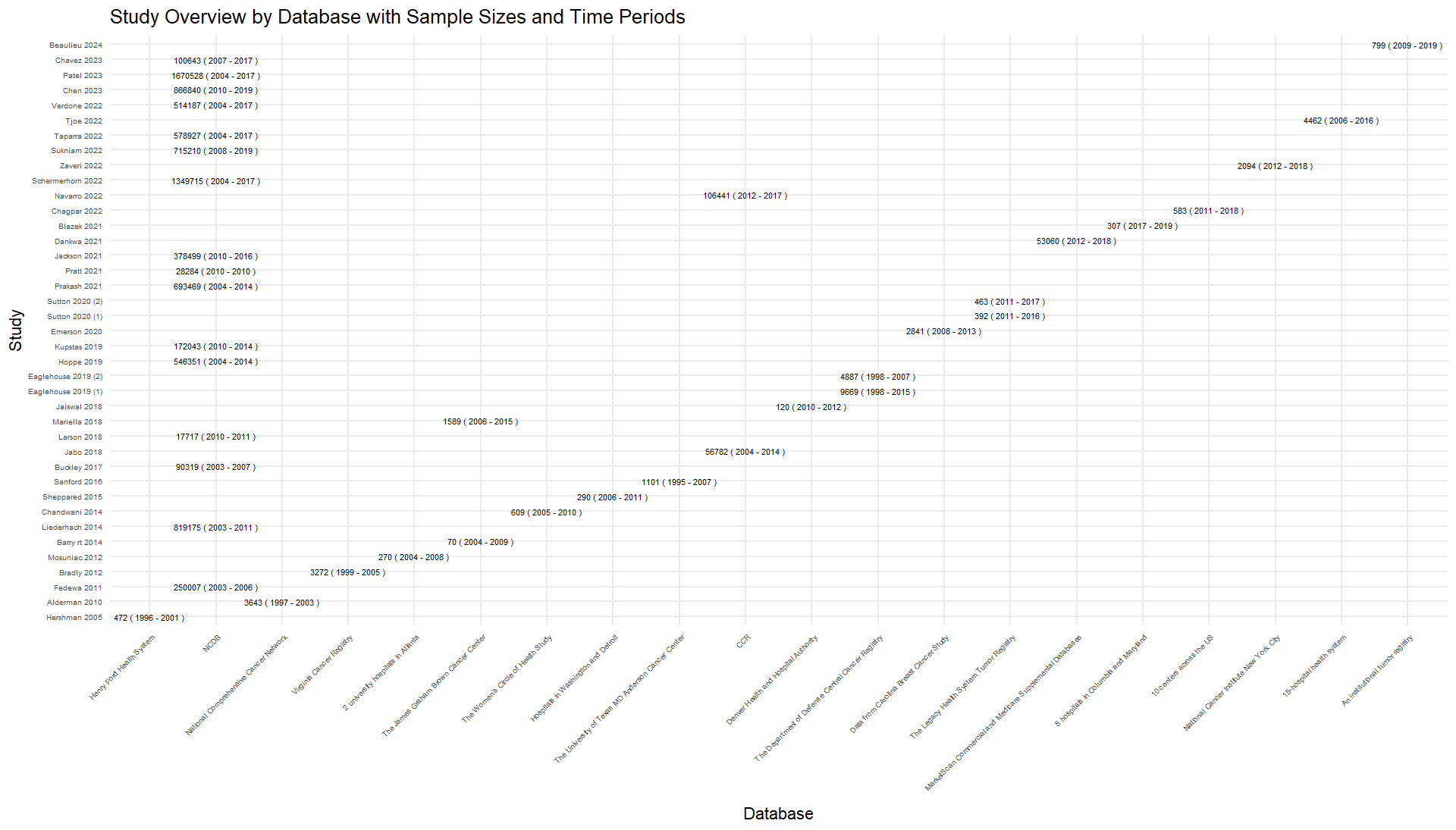


Appendix S2: R Code for the Finalized Summary DAG

#Load required Packages
library(dagitty)
library(ggdag)
## Attaching package: 'ggdag'

## The following object is masked from 'package:stats':
## filter

library(ggplot2)
set.seed(12143)
#summary of of all included studies: A composit DAG
dagRace <- dagify(O1 ~ RE + HRF + GS + A + C+ O2,
 O2 ~ RE + MD + DOT + Y + AI + PS + TPH + TD + SO + MS + SCH + IS + TCH + FCH + S + SBA + HD + ST + SFT + ES + PC + R + CP + CS + FI,
 PC ~ RE,
 R ~ RE,
 CP ~ RE,
 CS ~ RE,
 FI ~ RE,
 HD ~ RE,
 ST ~ RE,
 SFT ~ RE,
 ES ~ RE,
 IS ~ RE,
 TCH ~ RE,
 FCH ~ RE,
 S ~ RE,
 SBA ~ RE,
 TT ~ RE,
 exposure = "RE",
 outcome = c("O1", "O2"),
 labels = c(O1 = "Survival", O2 = "Time To Treatment",
 RE = "Racial/ethnic groups",
 HRF = "Health Risk Factors",
 GS = "Gender/Sex",
 MD = "Method of Diagnosis",
 DOT = "Delay in other Treatments",
 Y = "Year of Diagnosis",
 AI = "Access to Information",
 PS = "Performance Status",
 TPH = "Trust in Physician",
 TD = "Treatment Duration",
 SO = "Surgical Outcomes",
 MS = "Marital Status",
 SCH = "Surgeon Characteristics",
 IS = "Insurance Status",
 TCH = "Tumor Characteristics",
 FCH = "Facility Characteristics",
 SBA = "Structural Barrier/Access",
 HD = "HealthCare Discrimination",
 ST = "Surgery Type",
 SFT = "Surgery as first Treatment",
 ES = "English Speaking",
 PC = "Pretreatment Care",
 R = "Recurrence",
 CP = "Clinical Presentation",
 CS = "Cancer Stage",
 FI = "Financial Issues",
 A = "Age",
 C = "Comorbidities",
 S = "SES",
 TT = "Therapy Type"))

dag_plot <- ggdag_status(dagRace,var=c("O1","O2"),text_size = 2, text_col = "black",node_size=6, use_labels = "label") +
 theme_void()


print(dag_plot)
